# Genome-wide analysis of over 106 000 individuals identifies 9 neuroticism-associated loci

**DOI:** 10.1038/mp.2016.49

**Published:** 2016-04-12

**Authors:** D J Smith, V Escott-Price, G Davies, M E S Bailey, L Colodro-Conde, J Ward, A Vedernikov, R Marioni, B Cullen, D Lyall, S P Hagenaars, D C M Liewald, M Luciano, C R Gale, S J Ritchie, C Hayward, B Nicholl, B Bulik-Sullivan, M Adams, B Couvy-Duchesne, N Graham, D Mackay, J Evans, B H Smith, D J Porteous, S E Medland, N G Martin, P Holmans, A M McIntosh, J P Pell, I J Deary, M C O'Donovan

**Affiliations:** 1 Institute of Health and Wellbeing, University of Glasgow, Glasgow, UK; 2 MRC Centre for Neuropsychiatric Genetics and Genomics, Cardiff University, Cardiff, UK; 3 Centre for Cognitive Ageing and Cognitive Epidemiology, Department of Psychology, University of Edinburgh, Edinburgh, UK; 4 School of Life Sciences, College of Medical, Veterinary and Life Sciences, University of Glasgow, Glasgow, UK; 5 QIMR Berghofer Medical Research Institute, Herston, QLD, Australia; 6 Medical Research Council Human Genetics Unit, Institute of Genetics and Molecular Medicine, University of Edinburgh, Edinburgh, UK; 7 MRC Lifecourse Epidemiology Unit, University of Southampton, Southampton General Hospital, Southampton, UK; 8 Generation Scotland, Centre for Genomic and Experimental Medicine, Institute of Genetics and Molecular Medicine, University of Edinburgh, Edinburgh, UK; 9 Program in Medical and Population Genetics, Broad Institute of MIT and Harvard, Cambridge, MA, USA; 10 Analytical and Translational Genetics Unit, Department of Medicine, Massachusetts General Hospital and Harvard Medical School, Boston, MA, USA; 11 Stanley Center for Psychiatric Research, Broad Institute of MIT and Harvard, Cambridge, MA, USA; 12 Division of Psychiatry, University of Edinburgh, Edinburgh, UK; 13 Division of Population Health Sciences, University of Dundee, Dundee, UK; 14 Medical Genetics Section, Centre for Genomic and Experimental Medicine, Institute of Genetics and Molecular Medicine, University of Edinburgh, Edinburgh, UK

## Abstract

Neuroticism is a personality trait of fundamental importance for psychological well-being and public health. It is strongly associated with major depressive disorder (MDD) and several other psychiatric conditions. Although neuroticism is heritable, attempts to identify the alleles involved in previous studies have been limited by relatively small sample sizes. Here we report a combined meta-analysis of genome-wide association study (GWAS) of neuroticism that includes 91 370 participants from the UK Biobank cohort, 6659 participants from the Generation Scotland: Scottish Family Health Study (GS:SFHS) and 8687 participants from a QIMR (Queensland Institute of Medical Research) Berghofer Medical Research Institute (QIMR) cohort. All participants were assessed using the same neuroticism instrument, the Eysenck Personality Questionnaire-Revised (EPQ-R-S) Short Form's Neuroticism scale. We found a single-nucleotide polymorphism-based heritability estimate for neuroticism of ∼15% (s.e.=0.7%). Meta-analysis identified nine novel loci associated with neuroticism. The strongest evidence for association was at a locus on chromosome 8 (*P*=1.5 × 10^−15^) spanning 4 Mb and containing at least 36 genes. Other associated loci included interesting candidate genes on chromosome 1 (*GRIK3* (*glutamate receptor ionotropic kainate 3*)), chromosome 4 (*KLHL2* (*Kelch-like protein 2*)), chromosome 17 (*CRHR1* (*corticotropin-releasing hormone receptor 1*) and *MAPT* (*microtubule-associated protein Tau*)) and on chromosome 18 (*CELF4* (*CUGBP elav-like family member 4*)). We found no evidence for genetic differences in the common allelic architecture of neuroticism by sex. By comparing our findings with those of the Psychiatric Genetics Consortia, we identified a strong genetic correlation between neuroticism and MDD and a less strong but significant genetic correlation with schizophrenia, although not with bipolar disorder. Polygenic risk scores derived from the primary UK Biobank sample captured ∼1% of the variance in neuroticism in the GS:SFHS and QIMR samples, although most of the genome-wide significant alleles identified within a UK Biobank-only GWAS of neuroticism were not independently replicated within these cohorts. The identification of nine novel neuroticism-associated loci will drive forward future work on the neurobiology of neuroticism and related phenotypes.

## Introduction

Neuroticism is a dimension of personality that has been studied for ∼100 years, is present in most personality trait theories and questionnaires and is found in the lexicons of most human cultures.^1^ Individual differences in neuroticism are highly stable across the life course.^2^ Higher neuroticism is associated with considerable public health and economic costs,^3^ premature mortality^4^ and a range of negative emotional states and psychiatric disorders, including major depressive disorder (MDD), anxiety disorders, substance misuse disorders, personality disorders and schizophrenia.^5, 6, 7, 8, 9^ Thus, the study of neuroticism is not only important for understanding an important dimension of personality but may also illuminate the aetiology of a range of psychiatric disorders.^10, 11^

Eysenck^12^ suggested a biological basis for neuroticism over 50 years ago. Although the biological underpinnings of personality traits are not understood, genetic factors are clearly involved. Twin studies suggest that ∼40% of the trait variance for neuroticism is heritable,^2, 13, 14, 15, 16, 17^ of which between 15 and 37% is explained by variation in common single-nucleotide polymorphisms (SNPs)^17, 18^ and is potentially detectable using the genome-wide association study (GWAS) paradigm. The clear links between neuroticism, psychopathology and other adverse health outcomes—and the implications for global health that would result from a better understanding of its mechanisms^19^—provide a strong rationale for large-scale GWAS to identify its genetic architecture (and genetic aetiology).

To date, individual GWASs of neuroticism have been limited by modest sample sizes and have delivered equivocal findings. Large meta-analyses of GWASs have also delivered modest findings. The recent Genetics of Personality Consortium (GPC) meta-analysis of neuroticism, which included 73 447 individuals from 29 discovery cohorts plus a replication cohort, identified only one genome-wide significant associated locus, at *MAGI1* on chromosome 3 (*P*=2.38 × 10^−8^).^18^ Within two of the cohorts in this GPC study, common genetic variants explained ∼15% of the variance in neuroticism.^18^

In our study, seeking additional associated loci, we have conducted a meta-analysis that included GWAS results from the UK Biobank cohort, the Generation Scotland: Scottish Family Health Study (GS:SFHS) cohort^20^ and the QIMR (Queensland Institute of Medical Research) Berghofer Medical Research Institute Study in Adults (QIMR) cohort.^2, 13, 14^ The UK Biobank is the largest single GWAS sample of neuroticism to date and probably the most homogeneous in terms of ascertainment strategy and assessment methodology. In addition, we evaluated the genetic relationship between neuroticism and three major psychiatric phenotypes for which there are large, publically accessible GWAS data sets: MDD, schizophrenia and bipolar disorder (BD). Finally, we have compared our findings with those from the GPC meta-analytic GWAS of neuroticism,^18^ as well as the CONVERGE consortium for MDD.^21^

## Materials and methods

### Sample

UK Biobank is a large prospective cohort of more than 502 000 residents of the United Kingdom, aged between 40 and 69 years.^22^ The aim of UK Biobank aim is to study the genetic, environmental, medication and lifestyle factors that cause or prevent disease in middle and older age. Recruitment occurred over a 4-year period from 2006 to 2010. Baseline assessments included social, cognitive, personality (the trait of neuroticism), lifestyle and physical health measures. For the present study, we used the first genetic data release (June 2015) based on approximately one-third of UK Biobank participants. Aiming to maximise homogeneity, we restricted the sample to those who reported being of white UK ancestry and for whom neuroticism phenotype data were available (*n*=91 370).

We also made use of data provided by investigators from the GS:SFHS^20^ and QIMR cohorts^2, 13, 14^ to conduct a meta-analysis based on samples for which we could readily access individual genotypes and which were assessed using the same measure of neuroticism. The GS:SFHS sample comprised 7196 individuals and the QIMR sample comprised 8687 individuals. Individuals (*n*=537) who had participated in both UK Biobank and GS:SFHS were removed from the GS:SFHS sample based on relatedness checking using the genetic data.

Note that we were unable to incorporate the published data from the GPC as the neuroticism measure used in that study was derived from an item response theory analysis (prohibiting inverse variance-weighted meta-analysis due to the differences in variance and heterogeneity of the measure). In addition, there was no information on the sample size for each SNP (prohibiting sample size-weighted meta-analysis) and the majority of participants in the QIMR cohort were included within the GPC meta-analysis.

This study obtained informed consent from all participants and was conducted under generic approval from the National Health Service (NHS) National Research Ethics Service (approval letter dated 17 June 2011, Ref 11/NW/0382) and under UK Biobank approvals for application 6553 ‘Genome-wide association studies of mental health' (principal investigator Daniel Smith) and 4844 ‘Stratifying Resilience and Depression Longitudinally' (principal investigator Andrew McIntosh).

### Neuroticism phenotype

Neuroticism was assessed in all three cohorts (UK Biobank, GS:SFHS and QIMR) using the 12 items of the neuroticism scale from the Eysenck Personality Questionnaire-Revised Short Form (EPQ-R-S)^23^ (Supplementary Table S1). Respondents answered ‘yes' (score 1) or ‘no' (score 0) to each of the questions, giving a total neuroticism score for each respondent of between 0 and 12. This short scale has a reliability of more than 0.8 (ref. 23) and high concurrent validity; for example, in a sample of 207 older people EPQ-R-S scores correlated 0.85 with the neuroticism score from the NEO-Five Factor Inventory, the scale most widely used internationally.^24, 25^

### Genotyping and imputation

In June 2015, UK Biobank released the first set of genotype data for 152 729 UK Biobank participants. Approximately 67% of this sample was genotyped using the Affymetrix UK Biobank Axiom array (Santa Clara, CA, USA) and the remaining 33% were genotyped using the Affymetrix UK BiLEVE Axiom array. These arrays have over 95% content in common. Only autosomal data were available under the current data release. Data were pre-imputed by UK Biobank as fully described in the UK Biobank interim release documentation.^26^ Briefly, after removing genotyped SNPs that were outliers or were multiallelic or of low frequency (minor allele frequency (MAF) <1%), phasing was performed using a modified version of SHAPEIT2 and imputation was carried out using IMPUTE2 algorithms, as implemented in a C++ platform for computational efficiency.^27, 28^ Imputation was based upon a merged reference panel of 87 696  888 biallelic variants on 12 570 haplotypes constituted from the 1000 Genomes Phase 3 and UK10K haplotype panels.^29^ Variants with MAF <0.001% were excluded from the imputed marker set. Stringent quality control before release was applied by the Wellcome Trust Centre for Human Genetics, as described in UK Biobank documentation.^30^

### Statistical analysis

#### Quality control and association analyses

Before all analyses, further quality control measures were applied. Individuals were removed based on UK Biobank genomic analysis exclusions (Biobank Data Dictionary item #22010), relatedness (#22012: genetic relatedness factor; a random member of each pair of individuals with KING-estimated kinship coefficient >0.0442 was removed), gender mismatch (#22001: genetic sex), ancestry (#22006: ethnic grouping; principal component (PC) analysis identified probable Caucasians within those individuals who were self-identified as British and other individuals were removed from the analysis) and quality control failure in the UK BiLEVE study (#22050: UK BiLEVE Affymetrix quality control for samples and #22051: UK BiLEVE genotype quality control for samples). A sample of 112 031 individuals remained for further analyses. Of these, 91 370 had neuroticism scores. Genotype data were further filtered by removal of SNPs with Hardy–Weinberg equilibrium *P*<10^−6^, with MAF <0.01, with imputation quality score <0.4 and with data on <95% of the sample after excluding genotype calls made with <90% posterior probability, after which 8 268 322 variants were retained.

Association analysis was conducted using linear regression under a model of additive allelic effects with sex, age, array and the first 8 PCs (Biobank Data Dictionary items #22009.01 to #22009.08) as covariates. Genetic PCs were included to control for hidden population structure within the sample, and the first 8 PCs, out of 15 available in the Biobank, were selected after visual inspection of each pair of PCs, taking forward only those that resulted in multiple clusters of individuals after excluding individuals self-reporting as being of non-white British ancestry (Biobank Data Dictionary item #22006). The distribution of the neuroticism score was assessed for skewness and kurtosis (coefficients were 0.56 and −0.61, respectively) and found to be sufficiently ‘normal' (both coefficients are between −1 and 1) to permit analysis using linear regression. GWASs of neuroticism were additionally performed separately for females (*N*=47 196) and males (*N*=44174) using linear regression (as above), with age, array and the first 8 PCs as covariates.

#### Heritability, polygenicity and cross-sample genetic correlation

Univariate GCTA-GREML analyses were used to estimate the proportion of variance explained by all common SNPs for the neuroticism phenotype.^31^ We additionally applied linkage disequilibrium score regression (LDSR)^32^ to the summary statistics to estimate SNP heritability (*h*^2^_SNP_) and to evaluate whether inflation in the test statistics is the result of polygenicity or of poor control of biases such as population stratification. Genetic correlations between neuroticism scores in the three cohorts (UK Biobank, QIMR and GS:SFHS) were tested, and genetic correlations between neuroticism, schizophrenia, BD and MDD were evaluated in the UK Biobank sample using LDSR,^33^ a process that corrects for potential sample overlap without relying on the availability of individual genotypes.^32^ For the psychiatric phenotypes, we used GWAS summary statistics provided by the Psychiatric Genomics Consortium (http://www.med.unc.edu/pgc/).^34, 35, 36^

#### Polygenic risk score analyses in the QIMR and GS:SFHS samples

In the QIMR sample (*N*=8687 individuals), polygenic risk scores for neuroticism (PRS-N) based on the summary statistics from the UK Biobank GWAS were computed with PLINK 1.90 (version 3 September 2015, https://www.cog-genomics.org/plink2/), for *P-*value thresholds (*P*_T_) 0.01, 0.05, 0.1, 0.5 and 1, following the procedure described by Wray *et al.*^37^ All subjects had GWAS data imputed to 1000G v.3 (http://csg.sph.umich.edu/abecasis/MaCH/download/). Only SNPs with a MAF ⩾0.01 and imputation quality *r*^2^ ⩾0.6 were used in the calculation of the PRS-N. Genotypes were LD pruned using clumping to obtain SNPs in approximate linkage equilibrium with an *r*^2^<0.1 within a 10 000 bp window. As QIMR participants were related, predictions were calculated using GCTA (Genome-wide Complex Trait Analysis, version 1.22),^38^ using the following linear mixed model: EPQ-N=intercept+β0 × covariates+β2 × *g*+*e* with *g*~*N*(0, GRM), where EPQ is neuroticism measured by EPQ (standardised sum score); covariates are age, sex, imputation chip, 10 genetic PCs and the standardised PRS (*P*_T_ 0.01, 0.05, 0.1, 0.5 or 1); *e* is error; and GRM is genetic relationship matrix. *P*-values were calculated using the *t*-statistic on the basis of the β and s.e. from the GCTA output. Variance explained by the PRS was calculated using: var(*x*) × *b*^2^/var(*y*), where *x* is the PRS, *b* is the estimate of the fixed effect from GCTA and *y* is the phenotype.

In the GS:SFHS sample, PRS-N based on the UK Biobank neuroticism GWAS results were created using PRSice from observed genotypes in 7196 individuals.^20, 39^ SNPs with a MAF <0.01 were removed before creating PRS-N. Genotypes were LD pruned using clumping to obtain SNPs in linkage equilibrium with an *r*^2^<0.25 within a 200-kb window. As above, five PRS-N were created containing SNPs according to the significance of their association with the phenotype, with *P*_Ts_ of 0.01, 0.05, 0.1, 0.5 and 1 (all SNPs). Linear regression models were used to examine the associations between the PRS-N and neuroticism score in GS, adjusting for age at measurement, sex and the first 10 genetic PCs to adjust for population stratification. The false discovery rate method was used to correct for multiple testing across the PRS-N at all five thresholds.^40^

#### Meta-analysis

Inverse variance-weighted meta-analysis of UK Biobank, GS:SFHS and QIMR results was performed, restricted to variants present in all three samples, using the METAL package (http://www.sph.umich.edu/csg/abecasis/Metal). Data were available across all 3 studies for 7 207 648 of the original 8 268 322 variants from the UK Biobank analysis. The total sample size included in the meta-analysis was *N*=106 716 (UK Biobank *N*=91 370; GS:SFHS *N*=6659; and QIMR *N*=8687).

## Results

### Neuroticism phenotype within UK Biobank and sociodemographic characteristics

**Figure 1:**
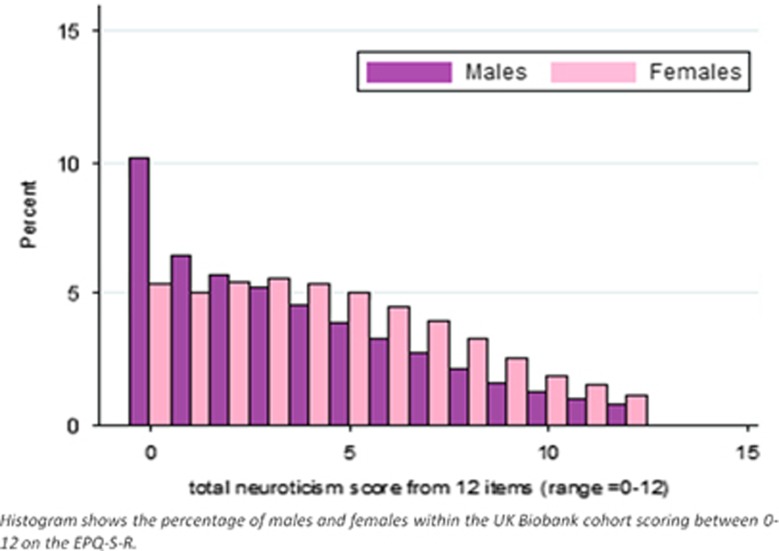
Distribution of neuroticism scores in the UK Biobank sample (*n*=91 370).

Sociodemographic details of the 91 370 UK Biobank participants used in this analysis, as well as the full UK Biobank cohort, are provided in Table 1 and the distributions of neuroticism scores for males and females in our sample are provided in Figure 1. The proportion of the UK Biobank neuroticism GWAS sample holding a degree was 31.4%, and the mean age of leaving full-time education for those without a degree was 16.5 years. Those in the full UK Biobank sample who responded to the neuroticism questions tended to be better educated than those who did not (33.4% had an undergraduate degree versus 27.7% in nonresponders). As expected,^41^ mean neuroticism scores were lower for men than for women (men mean EPQ-R-S=3.58, s.d.=3.19; women mean EPQ-R-S=4.58, s.d.=3.26; *P*=0.001). PC analysis of the 12 EPQ-R-S items showed that all items loaded highly on a single component, and the internal consistency (Cronbach's α) coefficient was 0.84 (Supplementary Table S2). Analysis of the entire UK Biobank sample (*N* with data=401 695) gave very similar results (Supplementary Table S2), suggesting the subsample analysed here is representative of the whole UK Biobank cohort.

### Meta-analysis of UK Biobank, GS:SFHS and QIMR samples

**Figure 2:**
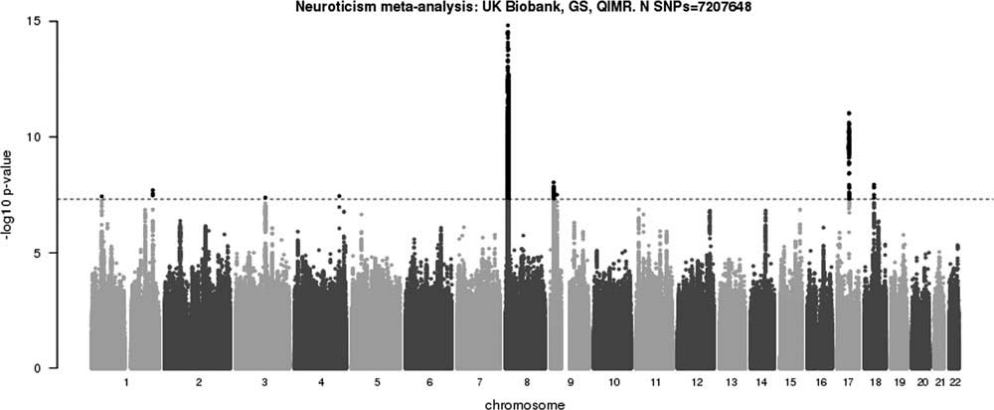
Manhattan plot of meta-analysis of GWAS from UK Biobank, GS:SFHS and QIMR samples (combined *N*=106 716). GS:SFHS, Generation Scotland: Scottish Family Health Study; GWAS, genome-wide association study; QIMR, Queensland Institute of Medical Research (QIMR) Berghofer Medical Research Institute.

In the combined data set, we obtained genome-wide significance for 9 independent loci: on chromosome 1 (two loci), chromosome 3, chromosome 4, chromosome 8, chromosome 9 (two loci), chromosome 17 and chromosome 18 (Figure 2 and Tables 2a and b).

Full details are provided in Tables 2a and b and the associated regions are depicted graphically as region plots in Supplementary Figures S3a–i. Candidate genes of particular note mapping to the associated loci include: the glutamatergic kainate receptor *GRIK3* (Supplementary Figure S3a);^42, 43^ *CELF4*, which regulates excitatory neurotransmission (Supplementary Figure S3i);^44^ and *CRHR1*, encoding corticotropin-releasing hormone receptor 1 (Supplementary Figure S3h), a protein that is central to the stress response.^45^ Associated loci are considered in greater detail within the discussion.

### Genome-wide association results in UK Biobank

Genome-wide association results from the UK Biobank cohort are summarised in Supplementary Materials: Supplementary Figure S1 (QQ plot); Supplementary Figure S2 (Manhattan plot); and Supplementary Table S3 (genome-wide significant loci associated with neuroticism).

Overall, the GWAS data showed modest deviation in the test statistics compared with the null (λ_GC_=1.152); this was negligible in the context of sample size (λ_GC_1000=1.003) (Supplementary Figure S1). LDSR^32^ suggested that deviation from the null was due to a polygenic architecture in which *h*^2^_SNP_ accounted for ∼14% of the population variance in neuroticism (liability scale *h*^2^_SNP_=0.136 (s.e. 0.0153)), rather than inflation due to unconstrained population structure (LD regression intercept=0.982 (s.e. 0.014)). Estimates of heritability using GCTA were similar to those using LD score regression (*h*^2^=0.156, s.e.=0.0074).

We observed a total of 8 independent loci exhibiting genome-wide significant associations with neuroticism (Supplementary Figure S2 and Supplementary Table S3) with the strongest evidence for association coming from a locus on chromosome 8 (*P*=1.02 × 10^−15^) at which there is an extensive LD block spanning 4 Mb (attributable to an inversion polymorphism that has suppressed recombination) containing at least 36 genes. Similar findings to those from the UK Biobank data set in a GWAS primarily assessing the genetics of well-being have also recently been posted in a non-peer-reviewed format.^46^

### Stratification by sex in UK Biobank

Neuroticism scores are in general higher in women than in men and it has been postulated that neuroticism may play a stronger aetiologic role in MDD in women than in men,^41, 47^ potentially explaining the greater prevalence of depressive and anxiety disorders in women.^48^ This suggests the possibility of sex-related genetic heterogeneity. We therefore conducted secondary analyses looking for sex-specific neuroticism loci in women (*N*=47 196) and men (*N*=44 174) respectively. To minimise heterogeneity, this analysis was restricted to the UK Biobank samples. SNP heritability (measured by LDSR) for each sex was comparable (female *h*^2^_SNP_=0.149 (s.e.=0.0169); male *h*^2^_SNP_=0.135 (s.e.=0.0237)), and was highly correlated between the sexes (genetic correlation=0.911 (s.e.=0.07); *P*=1.07 × 10^−38^) at a level that was not significantly different from 1 (*P*=0.21). In both sexes separately, the chromosome 8 locus was associated at genome-wide significance but no other single locus attained significance. Overall, we found no evidence for genetic differences in the common allelic architecture of neuroticism by sex.

### Genetic correlation of neuroticism with MDD, schizophrenia and BD

LDSR showed strong genetic correlation between neuroticism and MDD (genetic correlation= 0.64, s.e.=0.071, *P*=3.31 × 10^−19^) and a smaller, but significant, correlation between neuroticism and schizophrenia (genetic correlation=0.22, s.e.=0.05, *P*=1.96 × 10^−05^) (Table 3). We found no significant overlap between neuroticism and BD (genetic correlation=0. 07, s.e.=0.05, *P*=0.15). Similar results based solely on the UK Biobank data set have been reported recently in a non-peer-reviewed format.^49^

### Genetic correlations for neuroticism between UK Biobank, GS:SFHS and QIMR samples

The LDSR-calculated genetic correlation for neuroticism between the three samples was strong: between UK Biobank and GS:SFHS the genetic correlation was 0.91 (s.e.=0.15, *P*=4.04 × 10^−09^); between UK Biobank and QIMR the genetic correlation was 0.74 (s.e.=0.14, *P*=2.49 × 10^−07^); and between GS:SFHS and QIMR the genetic correlation was 1.16 (s.e.=0.35, *P*=0.0009). Note that the true maximum for a genetic correlation is bounded by 1. That the LD score estimate is greater than this reflects the imprecision in the estimate as indicated by the large s.e., in the context of which we interpret this as evidence for high but imprecisely estimated genetic correlation between the two samples.

### PRS analysis for neuroticism in GS:SFHS and QIMR samples

Table 4 shows the results of PRS analysis (based on the UK Biobank-only GWAS) within the GS:SFHS and QIMR samples. At all thresholds tested, PRS-N predicted neuroticism, although the amount of variance explained was small (at ∼1%).

### Comparison with findings from GPC meta-analysis

In contrast to the finding of the GPC meta-analysis, we did not identify a genome-wide significant association close to *MAGI1* within 3p14.^18^ However, within the UK Biobank sample, the same allele at the associated SNP from that study (rs35855737) did show a trend for association (β=0.035, s.e.=0.02, *P*=0.07; two tailed).

### Comparison with findings from the CONVERGE consortium study of MDD

The recently published CONVERGE consortium study of Chinese women with recurrent and melancholic MDD identified two loci contributing to risk of MDD on chromosome 10: one near the *SIRT1* gene (rs12415800; *P*=2.53 × 10^−10^) and the other in an intron of the *LHPP* gene (rs35936514, *P*=6.45 × 10^−12^).^21^ Neither of these index SNPs were associated with neuroticism within the UK Biobank sample (for rs12415800 β=−0.107, s.e.=0.066, *P*=0.1036, freq A=0.013; and for rs35936514 β=0.021, s.e.=0.0378, *P*=0.5832, freq T=0.041).

## Discussion

The identification of nine independent loci showing genome-wide significant associations with neuroticism within our combined meta-analysis represents a significant advance. In contrast, a recent meta-analysis of neuroticism conducted by the GPC (*n*=73 447) identified only a single genome-wide significant locus.^18^

There are several possible explanations for this difference. All three of the cohorts in our study used the same 12-item neuroticism assessment instrument (the EPQ-R-S), whereas the GPC study assessed neuroticism scores using different instruments across cohorts, with an item response theory approach to harmonise scores.^18^ Furthermore, the UK Biobank cohort is by far the largest sample ever studied for neuroticism genetics and all of the participants were of white British ethnicity, minimising population stratification and also addressing potential problems with cultural variation in the interpretation of neuroticism questionnaire items. In addition, quality control steps in the UK Biobank sample were performed in a single centre in a consistent way.

The most significant associated locus on chromosome 8, which was independently associated at genome-wide significance for both men and women, spans a 4-Mb region of extended LD (the result of an inversion polymorphism) containing at least 36 genes (Tables 2a and b and Supplementary Figure S3e). The extended LD at this locus means that identifying the specific genes responsible for the association is likely to prove challenging. As an initial attempt to resolve the signal, we queried the index SNP (rs12682352) at the BRAINEAC (http://www.braineac.org/) brain expression quantitative trait locus resource. This identified *ERI1* as the only protein coding gene within the locus whose expression was associated with the index SNP in brain, but only nominally so (*P*=0.019) and not at a level that would reliably point to this gene as likely explaining the association.

The locus on chromosome 17 (rs111433752 at 43.8 Mb; Supplementary Figure S3h) similarly maps to an inversion polymorphism spanning multiple genes and therefore we cannot attribute the association to any particular gene. As with the locus on chromosome 8, inspection of expression quantitative trait loci in the region in BRAINEAC did not help to resolve the signal. Nevertheless, this locus contains a notable candidate gene, *CRHR1*, encoding corticotropin-releasing hormone receptor 1. In the presence of corticotropin-releasing hormone, *CRHR1* triggers the downstream release of the stress response-regulating hormone cortisol. CRHR1 is therefore a key link in the hypothalamic–pituitary–adrenal pathway that mediates the body's response to stress and that is abnormal in severe depression.^45^ *CRHR1 per se* has also been shown to be involved in anxiety-related behaviours in mice and has also been genetically associated with panic disorder in humans.^50^

Another potential candidate gene within the extended region of genome-wide significant association at the chromosome 17 locus is *MAPT* that encodes the microtubule-associated protein Tau. There is evidence that Tau is present in the postsynaptic compartment of many neurons^51^ and *MAPT* knockout in mice leads to defects in hippocampal long-term depression,^52^ as well as mild network-level alterations in brain function.^53^ The clearest candidate gene at one of the other loci, *CELF4* on chromosome 18 at ∼35 Mb, encodes an mRNA-binding protein known to participate in a major switch in Tau protein isoform distribution after birth in the mammalian brain.^54^ CELF4 is expressed predominantly in glutamatergic neurones, and recent studies suggest it has a central role in regulating excitatory neurotransmission by modulating the stability and/or translation of a range of target mRNAs.^44^

The finding of an association with a locus on chromosome 1 (rs490647), which includes the glutamatergic kainate receptor *GRIK3*, is of considerable interest given that abnormalities of the glutamate system are implicated in the pathophysiology of MDD.^55, 56, 57, 58, 59, 60^ Furthermore, a recent glutamate receptor gene expression study in a large cohort of post-mortem subjects, including some individuals with MDD who had completed suicide, found *GRIK3* to be the strongest predictor of suicide.^43^

On chromosome 4, rs62353264 lies a short distance upstream of *KLHL2* that encodes a BTB-Kelch-like protein. KLHL2 is an actin-binding protein and has also been reported to be part of a complex that ubiquitinates NPTXR, the neuronal pentraxin receptor,^61^ among other targets. Expression of KLHL2 has been reported to be enriched in brain, and it is localised to cytoplasm and processes of neurons and astrocytes, being found at sites of ruffles and other actin network-containing membrane outgrowths.^62, 63^ The associated region at this locus is short (∼150 kb), and although several other genes lie within 500 kb of the peak association at this locus, none is as promising a candidate as *KLHL2*.

The associated region in chromosome 9p23 at ∼11.2–11.7 Mb contains no protein-coding genes; the nearest gene on the telomeric side, with its 5′-end located ∼650 kb from the associated region, is *PTPRD*. This gene encodes a receptor-type protein tyrosine phosphatase known to be expressed in brain and with an organising role at a variety of synapses,^64^ including those that play a role in synaptic plasticity. *PTPRD* is also known to harbour variation associated with restless legs syndrome.^65^ This is a credible candidate but particular caution is required given the distance between the associated locus and this gene.

In addition to identifying genome-wide significant loci, our study contributes further to understanding the genetic architecture of neuroticism and its relationship to other disorders. Our SNP-based heritability estimate for neuroticism was ∼0.15, as estimated using GCTA, and only slightly lower using LDSR. This is consistent with the estimates reported by the GPC^18^ in the two homogeneous subsets of the data they tested, and considerably greater than some earlier reports of ∼6%.^66, 67^ Despite differences in the distribution of neuroticism by sex, SNP-based heritability was similar for both men and women and the genetic correlation between sexes was not significantly different from 1, suggesting a similar common variant architecture for both, and that differences in trait scores between the sexes are likely to result from structural variants, rare alleles and/or environmental exposures.

PRS analysis of neuroticism within the GS:SFHS and QIMR samples supported the expected highly polygenic architecture of neuroticism; despite the large discovery UK Biobank sample—but consistent with the modest number of GWS findings identified in this large sample—extremely weakly associated alleles at relaxed association thresholds (for example, *P*_T_ up to at least 0.5) contributed to the variance captured by the signal.

Consistent with current practice, we regard the meta-analysis results as the primary outputs of this study. However, it is notable that although the results of the polygenic risk score analyses show that *en masse*, alleles that associate with neuroticism in UK Biobank tend to do the same in those with higher neuroticism within GS:SFHS and QIMR, this is not evident for the loci attaining genome-wide significance. It should be noted that most of the associated alleles identified from the UK Biobank GWAS were not independently replicated within the GS:SFHS and QIMR cohorts, nor within the large Genetics of Personality Consortium meta-analysis. Of the eight loci that were genome-wide significant in the UK Biobank data set, only five were significant within the meta-analysis. With the exception of the locus on chromosome 17, none of these were replicated across the GS:SFHS and QIMR samples, and the most significantly associated locus, that on chromosome 8, is not significant in either sample (Supplementary Table S4). The large standard errors for the estimates of effect sizes in GS:SFHS and QIMR are consistent with low power of these population samples to detect loci (with the effect sizes seen in complex traits), and with the fact that fully independent replication (or refutation) will require much larger samples.

By comparing the overall association analysis results in our study with those from the Psychiatric Genomics Consortia, we identified a strong genetic correlation between neuroticism and MDD (0.64), and a weaker but still significant genetic correlation with schizophrenia (0.22), although not with BD. These findings are line with evidence suggesting that neuroticism and MDD—as well as, to a lesser extent, neuroticism and schizophrenia—share genetic risk factors in common.^68^ However, the present findings do not distinguish between a direct causal link between neuroticism and those other disorders^5, 7, 8, 69^ versus pleiotropy, whereby a proportion of risk alleles that influence neuroticism also exert an effect on the clinical diagnoses. Nevertheless, our findings suggest neuroticism as a potentially fruitful measure for efforts such as the Research Domain Criteria (RDoC) initiative that seek to use fundamental and quantitative characteristics to investigate the aetiology of psychiatric disorders across traditional nosological boundaries in order to develop a more biologically informed system of psychiatric classification.^70^

Our findings are of interest in the context of the limited success to date of GWAS studies of MDD. A recent mega-analysis of genome-wide association studies for MDD (9240 MDD cases and 9519 controls in the discovery phase, and 6783 MDD cases and 50 695 controls in the replication phase) failed to identify any genome-wide significant SNPs, suggesting that much larger samples are required to detect genetic effects for complex traits such as MDD.^36^ Given the high genetic correlation between neuroticism and MDD, combining the two data sets in a meta-analysis may be a plausible strategy to optimise the power of population samples in the search for a proportion of MDD loci, although noting that the two phenotypes are not perfectly genetically correlated. The MDD loci identified in a recent study of Chinese women with recurrent (*N*=5303) and melancholic (*N*=4509) MDD by the CONVERGE consortium^21^ did not overlap with any of the loci reported here; given the apparent modest power to detect genome-wide significant loci in our sample, population differences between the studies and substantial differences between the phenotypes, the absence of overlap does not provide any evidence against the validity of the CONVERGE study finding. Given that neuroticism is a personality trait established as phenotypically and genetically strongly associated with MDD, the identification of several new genome-wide significant loci for neuroticism represents an important potential entry point into the biology of MDD.

## Conclusion

Overall, our findings confirm a polygenic basis for neuroticism and substantial shared genetic architecture between neuroticism and MDD, and to a lesser extent with schizophrenia, though not with BD disorder. The identification of nine new loci associated with neuroticism represents a significant advance in this field and will drive future work on the neurobiology of a personality trait that has fundamental importance to human health and well-being.

## Figures and Tables

**Table 1:** Sociodemographic characteristics in UK Biobank

	*Full UK Biobank sample (*N=*502 665)*	*UK Biobank neuroticism GWAS sample (*N=*91 370)*
Age in years, mean (s.d.)	56.5 (8.1)	56.7 (7.9)
Age range (years)	37–73	40–73
Female, *N* (%)	273 472 (54.4)	47 196 (51.7)
Neuroticism score, mean (s.d.)	4.12 (3.3)	4.10 (3.3)
Undergraduate degree, *N* (%)	162 026 (32.2)	28 727 (31.4)
Age when left full-time education (for those without an undergraduate degree), mean (s.d.)	16.4 (3.5)	16.5 (2.8)

Abbreviation: GWAS, genome-wide association study.

**Table 2A:** Genome-wide significant index SNPs. Combined meta-analysis of UK Biobank, GS:SFHS and QIMR data sets

*Index SNP*	*Chr*	*Position*	*A1/A2*	*Freq*	*β (s.e.)*	P	*Direction (UKBB-GS-QMIR)*	*Heter* P	*Associated region*	*Genes*
rs490647	1	37 242 743	A/G	0.227	0.092 (0.017)	3.8 × 10^−8^	+++	0.577	37 219 429–37 261 085	*GRIK3*
rs4653663	1	225 927 218	A/T	0.255	0.091 (0.016)	2.0 × 10^−8^	+++	0.097	225 899 639–225 947 638	*ENAH, SRP9*
rs12637928	3	110 184 749	A/T	0.490	−0.077 (0.014)	4.3 × 10^−8^	−−−	0.663	110 103 126–110 299 632	*PVRL3* (579KB distal)
rs62353264	4	166 085 805	A/T	0.986	−0.335 (0.061)	3.7 × 10^−8^	−−+	0.261	166 063 134–166 198 156	*TMEM192, KLHL2, MSMO1*
rs12682352	8	8 646 246	T/C	0.525	0.115 (0.014)	1.5 × 10^−15^	+++	0.366	8 301 794–10 831 868	More than 10 genes
rs12378446	9	11 369 213	T/C	0.791	0.100 (0.017)	9.4 × 10^−9^	+++	0.919	11 131 371–11 880 898	*PTRD* (650KB distal)
rs4977844	9	23 295 899	C/G	0.358	0.083 (0.015)	3.2 × 10^−8^	+++	0.367	23 291 526–23 340 616	*ELAVL2*
rs111433752	17	43 857 989	T/G	0.790	−0.120 (0.018)	9.3 × 10^−12^	−−−	0.068	43 463 493–44 865 603	More than 10 genes
rs1187264	18	35 289 647	C/G	0.136	0.118 (0.021)	1.2 × 10^−8^	+++	0.526	35 287 090–35 413 260	*CELF4*

Abbreviations: Chr, chromosome; Freq, frequency; GS:SFHS, Generation Scotland: Scottish Family Health Study; Heter, heterogeneity; QIMR, Queensland Institute of Medical Research (QIMR) Berghofer Medical Research Institute; SNP, single-nucleotide polymorphism.

Shown are linkage disequilibrium (LD)-independent genome-wide significant SNP associations for neuroticism (sorted by genomic position according to UCSC hg19/NCBI Build 37). Column A1/A2 has the SNP alleles, with the first allele (A1) the reference allele for the frequency and β columns. Frequency of allele 1 is calculated in the UK BioBank data set. Chr and Position denote the location of the index SNP. β Is linear regression coefficient for allele1, and s.e. is the standard error for β. Associated region indicates range positions of SNPs with *r*^2^ >0.6 with the index and any other genome-wide association study (GWAS) significant SNP at the locus. The final column indicates protein-coding reference sequence genes at the associated loci (see region plots in Supplementary Information) or where there are no genes at the associated locus, the nearest gene if <1 Mb from the locus.

**Table 2B:** Association results for genome-wide significant index SNPs in UK Biobank, GS:SFHS and QIMR data sets separately

*Index SNP*	*Chr*	*Position*	*UK Biobank*				*GS:SFHS*				*QIMR*			
			*β*	*s.e.*	P	*FRQ*	*β*	*s.e.*	P	*FRQ*	*β*	*s.e.*	P	*FRQ*
rs490647	1	37 242 743	0.088	0.018	7.79 × 10^−7^	0.227	0.073	0.065	0.257	0.234	0.157	0.066	0.017	0.243
rs4653663	1	225 927 218	0.079	0.017	5.12 × 10^−6^	0.255	0.117	0.062	0.060	0.260	0.219	0.064	0.001	0.259
rs12637928	3	110 184 749	−0.074	0.015	8.76 × 10^−7^	0.490	−0.073	0.055	0.186	0.506	−0.128	0.058	0.027	0.491
rs62353264	4	166 085 805	−0.335	0.065	2.36 × 10^−7^	0.986	−0.547	0.219	0.012	0.984	0.059	0.298	0.842	0.988
rs12682352	8	8 646 246	0.120	0.015	1.02 × 10^−15^	0.525	0.0005	0.111	0.997	0.539	0.063	0.057	0.265	0.528
rs12378446	9	11 369 213	0.100	0.019	9.69 × 10^−8^	0.791	0.123	0.068	0.071	0.793	0.084	0.070	0.233	0.784
rs4977844	9	23 295 899	0.083	0.016	2.02 × 10^−7^	0.358	0.136	0.058	0.019	0.351	0.018	0.060	0.767	0.352
rs111433752	17	43 857 989	−0.109	0.019	5.19 × 10^−9^	0.790	−0.143	0.073	0.050	0.806	−0.297	0.080	0.0002	0.788
rs1187264	18	35 289 647	0.123	0.022	2.36 × 10^−8^	0.136	0.029	0.081	0.720	0.136	0.131	0.083	0.113	0.132

Abbreviations: Chr, chromosome; FRQ, frequency; GS:SFHS, Generation Scotland: Scottish Family Health Study; QIMR, Queensland Institute of Medical Research (QIMR) Berghofer Medical Research Institute; SNP, single-nucleotide polymorphism.

**Table 3:** Genetic correlations between neuroticism and MDD, schizophrenia and bipolar disorder

	N *cases*	N *controls*	*Genetic correlation*	*s.e. genetic correlation*	*Significance (*P*-value)*
MDD	9240	9519	0.64	0.07	3.31 × 10^−19^
Bipolar disorder	7481	9250	0.07	0.05	0.1505
Schizophrenia	34 241	45 604	0.22	0.05	1.96 × 10^−5^

Abbreviation: MDD, major depressive disorder.

Columns ‘*N* cases' and ‘*N* controls' show the numbers of cases and controls in the corresponding PGC2 genome-wide association studies (https://www.med.unc.edu/pgc/downloads). Columns 4–6 present genetic correlation estimates, their s.e. and significance, respectively, calculated with linkage disequilibrium (LD) score regression tool (https://github.com/bulik/ldsc).

**Table 4:** Associations between the PRS for neuroticism based on the UK Biobank Neuroticism GWAS summary results, and neuroticism in GS:SFHS and QIMR samples, controlling for age, sex and 10 genetic principal components for population structure

*Threshold*	*β*	*s.e.*	*Percentage variance explained*	P-*value*	*Number of SNPs*
*GS:SFHS sample,* N=*7196*					
PRS<0.01	0.107	0.016	0.59	4.58 × 10^−11^	4531
PRS<0.05	0.123	0.014	1.00	5.27 × 10^−19^	15 533
PRS<0.1	0.131	0.013	1.30	3.23 × 10^−23^	27 216
PRS<0.5	0.132	0.012	1.48	3.45 × 10^−26^	95 552
PRS<1	0.131	0.012	1.46	6.93 × 10^−26^	146 088

*QIMR sample,* N=*8687*					
PRS<0.01	0.070	0.012	0.49	8.5 × 10^−09^	12 146
PRS<0.05	0.081	0.012	0.66	5.3 × 10^−12^	41 006
PRS<0.1	0.086	0.012	0.74	1.5 × 10^−13^	68,979
PRS<0.5	0.086	0.012	0.75	7.7 × 10^−14^	204 632
PRS<1	0.088	0.011	0.77	3.2 × 10^−14^	280 716

Abbreviations: GS:SFHS, Generation Scotland: Scottish Family Health Study; GWAS, genome-wide association study; PRS, polygenic risk scores; QIMR, Queensland Institute of Medical Research (QIMR) Berghofer Medical Research Institute; SNP, single-nucleotide polymorphism.

## Notes

JPP is a member of the UK Biobank Scientific Advisory Board and IJD and DJP were participants in UK Biobank. The other authors declare no conflict of interest.
