# Erratum: Genome-wide analysis of over 106 000 individuals identifies 9 neuroticism-associated loci

**DOI:** 10.1038/mp.2016.177

**Published:** 2016-09-13

**Authors:** D J Smith, V Escott-Price, G Davies, M E S Bailey, L Colodro-Conde, J Ward, A Vedernikov, R Marioni, B Cullen, D Lyall, S P Hagenaars, D C M Liewald, M Luciano, C R Gale, S J Ritchie, C Hayward, B Nicholl, B Bulik-Sullivan, M Adams, B Couvy-Duchesne, N Graham, D Mackay, J Evans, B H Smith, D J Porteous, S E Medland, N G Martin, P Holmans, A M McIntosh, J P Pell, I J Deary, M C O'Donovan

**Keywords:** Genome-wide association studies, Disease genetics, Population genetics, Neurological disorders

**Correction to:** *Molecular Psychiatry* **21,** 749–757; doi:10.1038/mp.2016.49

The GWAS of neuroticism conducted within the Queensland Institute of Medical Research (QIMR) Berghofer Medical Research Institute cohort did not include covariates of age, sex, genotyping batch and 10 principal components. Adding these covariates does not substantially change the pattern of results within the meta-analysis, but *P*-values for the nine reported loci have changed slightly (please see revised Figure 2, Table 2A and Table 2B). Of note is that, the *P*-value for the index SNP rs490647 on chromosome one is now 5.0 × 10^−8^ (previously 3.8 × 10^−8^) and the *P*-value for the index SNP rs62353264 on chromosome four is now 5.5 × 10^−8^ (previously 3.7 × 10^−8^).

**Figure 2:**
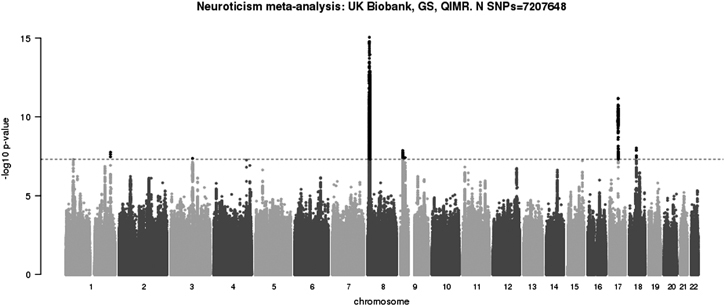
PowerPoint slide

**Table 2A:** Genome-wide significant index SNPs. Combined meta-analysis of UK Biobank, GS:SFHS and QIMR data sets

*Index SNP*	*Chr*	*Position*	*A1/A2*	*Freq*	*BETA (SE)*	P	*Direction (UKBB-GS-QMIR)*	*Heter* P	*Associated region*	*Genes*
rs490647	1	37 242 743	A/G	0.227	0.091 (0.017)	5.0 × 10^−8^	+++	0.720	37 219 429–37 261 085	*GRIK3*
rs4653663	1	225 927 218	A/T	0.255	0.091 (0.016)	1.8 × 10^−8^	+++	0.095	225 899 639–225 947 638	*ENAH, SRP9*
rs12637928	3	110 184 749	A/T	0.490	−0.077 (0.014)	4.3 × 10^−8^	−−−	0.695	110 103 126–110 299 632	*PVRL3* (579KB distal)
rs62353264	4	166 085 805	A/T	0.986	−0.330 (0.061)	5.5 × 10^−8^	−−+	0.158	166 063 134–166 198 156	*TMEM192, KLHL2, MSMO1*
rs12682352	8	8 646 246	T/C	0.525	0.115 (0.014)	9.0 × 10^−15^	+++	0.433	8 301 794–10 831 868	More than 10 genes
rs12378446	9	11 369 213	T/C	0.791	0.099 (0.017)	9.4 × 10^−9^	+++	0.831	11 131 371–11 880 898	*PTRD* (650KB distal)
rs4977844	9	23 295 899	C/G	0.358	0.083 (0.015)	1.4 × 10^−8^	+++	0.318	23 291 526–23 340 616	*ELAVL2*
rs111433752	17	43 857 989	T/G	0.790	−0.121 (0.018)	6.7 × 10^−12^	−−−	0.053	43 463 493–44 865 603	More than 10 genes
rs1187264	18	35 289 647	C/G	0.136	0.118 (0.021)	9.5 × 10^−9^	+++	0.515	35 287 090–35 413 260	*CELF4*

Abbreviations: Chr, chromosome; Freq, frequency; GS:SFHS, Generation Scotland: Scottish Family Health Study; Heter, heterogeneity; QIMR, Queensland Institute of Medical Research (QIMR) Berghofer Medical Research Institute; SNP, single-nucleotide polymorphism. Shown are linkage disequilibrium (LD)- independent genome-wide significant SNP associations for neuroticism (sorted by genomic position according to UCSC hg19/NCBI Build 37). Column A1/A2 has the SNP alleles, with the first allele (A1) the reference allele for the frequency and β columns. Frequency of allele 1 is calculated in the UK Biobank data set. Chr and Position denote the location of the index SNP. β is linear regression coefficient for allele1, and s.e. is the standard error for β. Associated region indicates range positions of SNPs with r^2^>0.6 with the index and any other genome-wide association study (GWAS) significant SNP at the locus. The final column indicates protein-coding reference sequence genes at the associated loci (see region plots in Supplementary Information) or where there are no genes at the associated locus, the nearest gene if <1 Mb from the locus.

**Table 2B:** Association results for genome-wide significant index SNPs in UK Biobank, GS:SFHS and QIMR datasets separately

*Index SNP*	*Chr*	*Position*	*UK Biobank*				*GS:SFHS*				*QIMR*			
			*BETA*	*s.e.*	P	*FRQ*	*BETA*	*s.e.*	P	*FRQ*	*BETA*	*s.e.*	P	*FRQ*
rs490647	1	37 242 743	0.088	0.018	7.79 × 10^−7^	0.227	0.073	0.065	0.257	0.234	0.139	0.065	0.031	0.243
rs4653663	1	225 927 218	0.079	0.017	5.12 × 10^−6^	0.255	0.117	0.062	0.060	0.260	0.217	0.062	0.0005	0.259
rs12637928	3	110 184 749	−0.074	0.015	8.76 × 10^−7^	0.490	−0.073	0.055	0.186	0.506	−0.123	0.056	0.028	0.491
rs62353264	4	166 085 805	−0.335	0.065	2.36 × 10^−7^	0.986	−0.547	0.219	0.012	0.984	0.147	0.291	0.612	0.988
rs12682352	8	8 646 246	0.120	0.015	1.02 × 10^−15^	0.525	0.0005	0.111	0.997	0.539	0.076	0.055	0.169	0.528
rs12378446	9	11 369 213	0.100	0.019	9.69 × 10^−8^	0.791	0.123	0.068	0.071	0.793	0.065	0.068	0.342	0.784
rs4977844	9	23 295 899	0.083	0.016	2.02 × 10^−7^	0.358	0.136	0.058	0.019	0.351	0.012	0.059	0.837	0.352
rs111433752	17	43 857 989	−0.109	0.019	5.19 × 10^−9^	0.790	−0.143	0.073	0.050	0.806	−0.301	0.078	0.0001	0.788
rs1187264	18	35 289 647	0.123	0.022	2.36 × 10^−8^	0.136	0.029	0.081	0.720	0.136	0.139	0.081	0.086	0.132

Abbreviations: Chr, chromosome; FRQ, frequency; GS:SFHS, Generation Scotland: Scottish Family Health Study; QIMR, Queensland Institute of Medical Research (QIMR) Berghofer Medical Research Institute; SNP, single-nucleotide polymorphism.

## PowerPoint slides

PowerPoint slide for Fig. 1

