# Supplementary figures and images for: Genome-wide analysis of over 106 000 individuals identifies 9 neuroticism-associated loci
Source: Mol Psychiatry. 2016 Apr 12;21(6):749–57. doi: 10.1038/mp.2016.49 (PMC4879189; doi:10.1038/mp.2016.49)

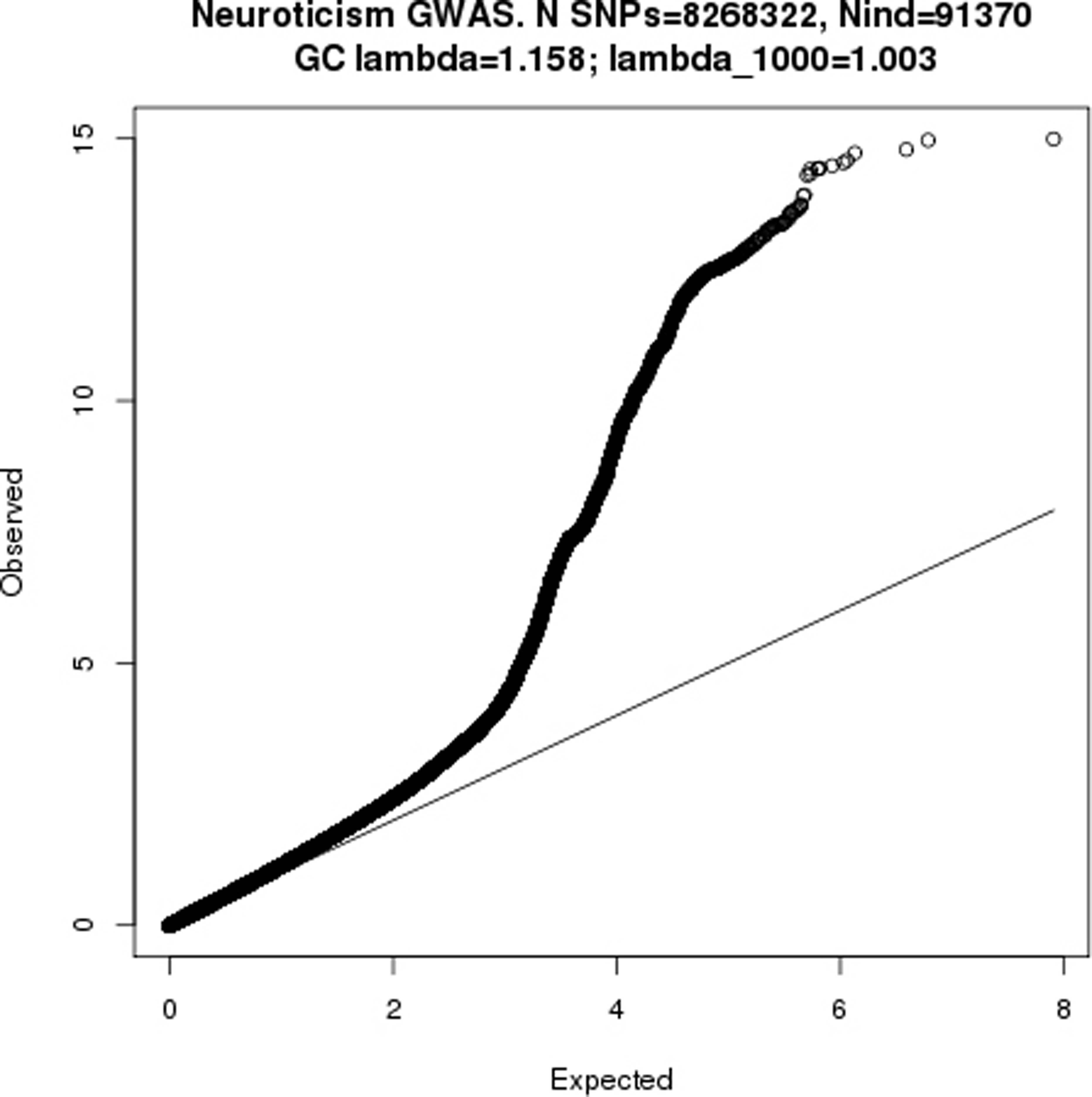

Supplement: Supplementary Figure 1 [file mp201649x1.tif]

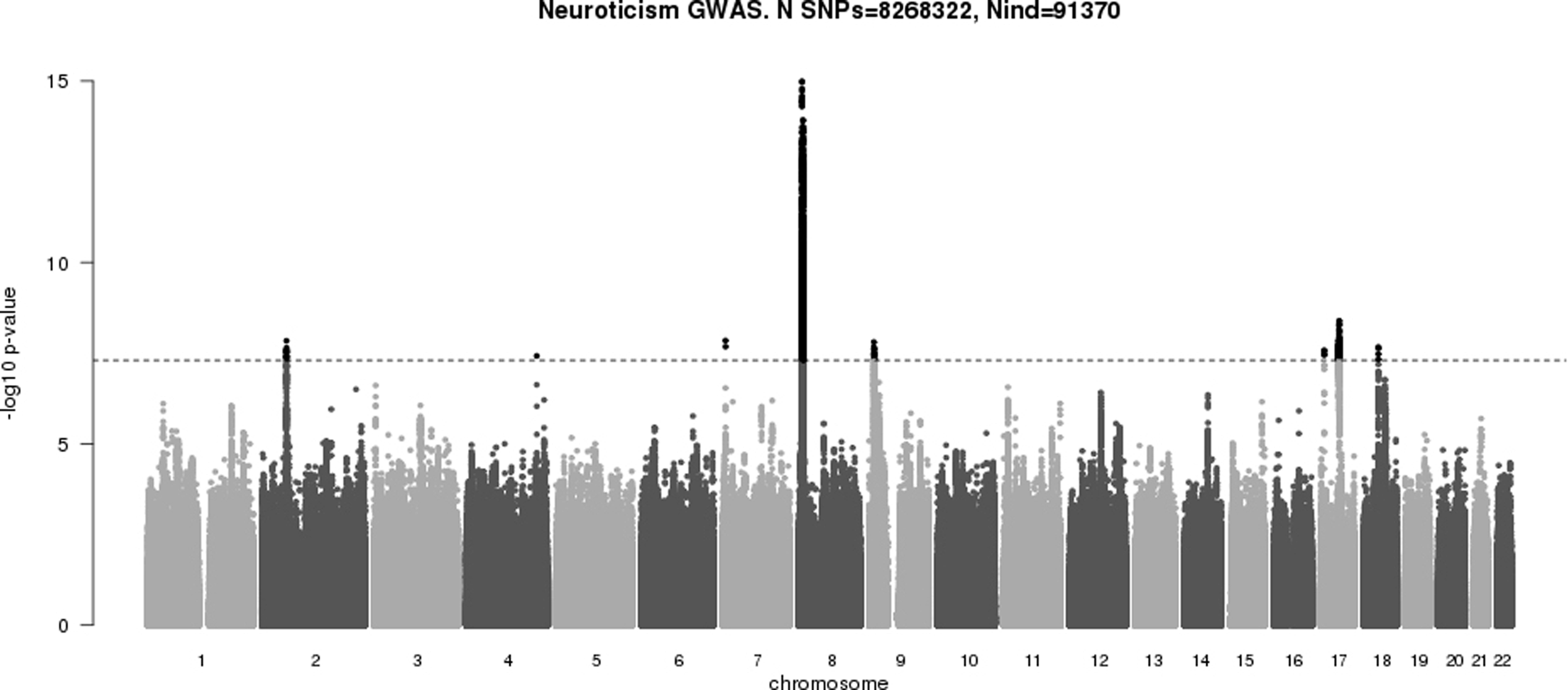

Supplement: Supplementary Figure 2 [file mp201649x2.tif]

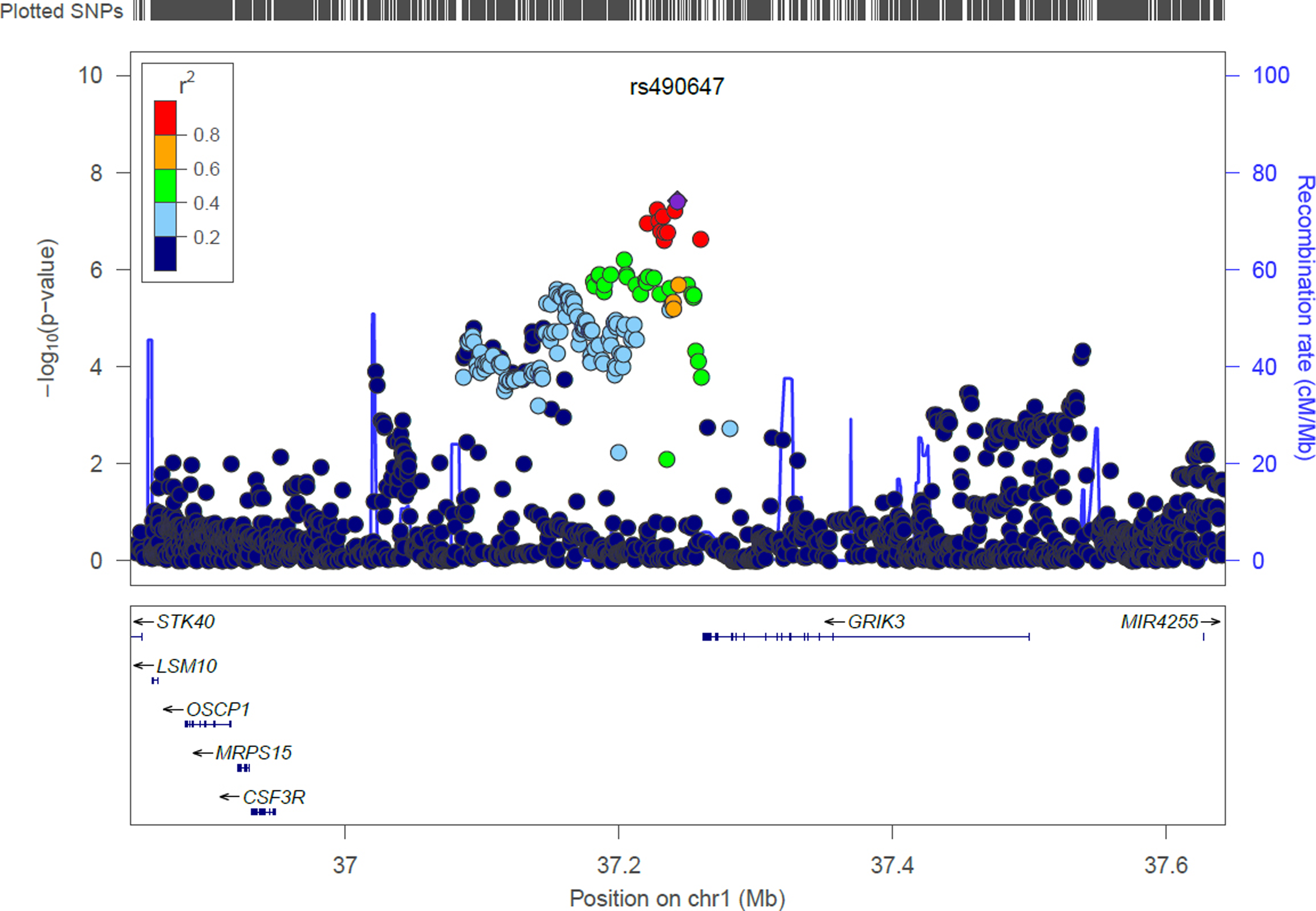

Supplement: Supplementary Figure 3 [file mp201649x3.tif]

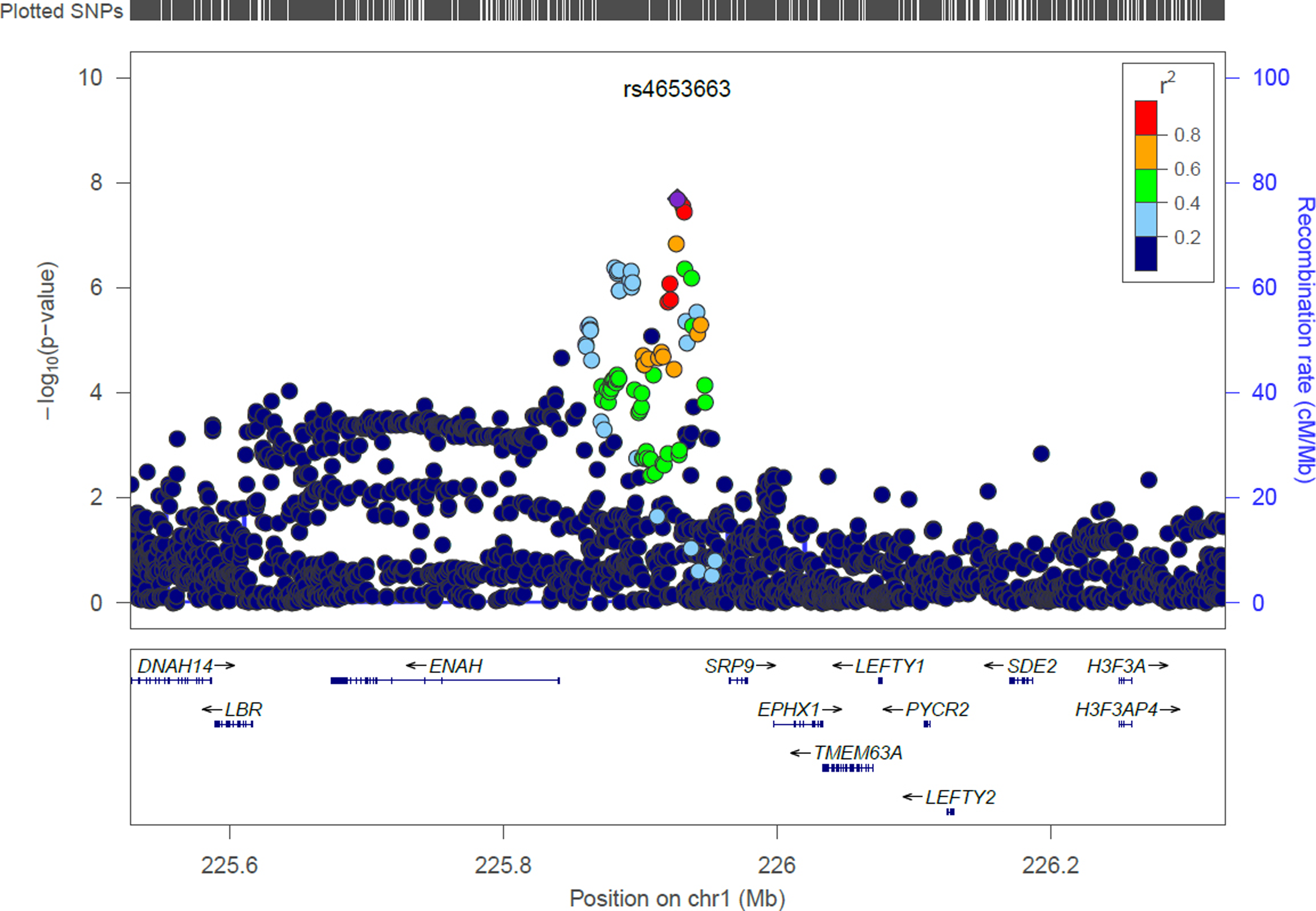

Supplement: Supplementary Figure 4 [file mp201649x4.tif]

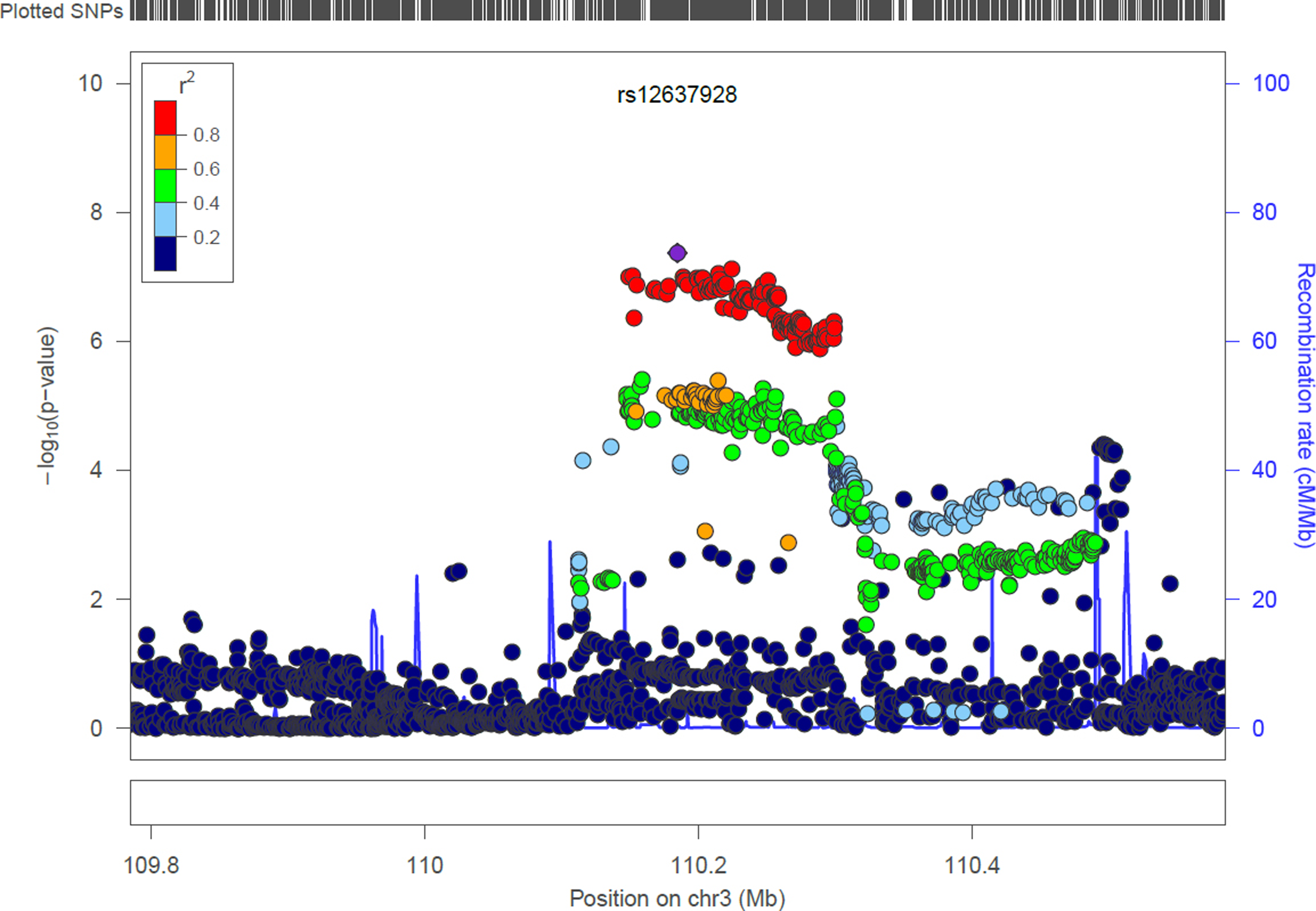

Supplement: Supplementary Figure 5 [file mp201649x5.tif]

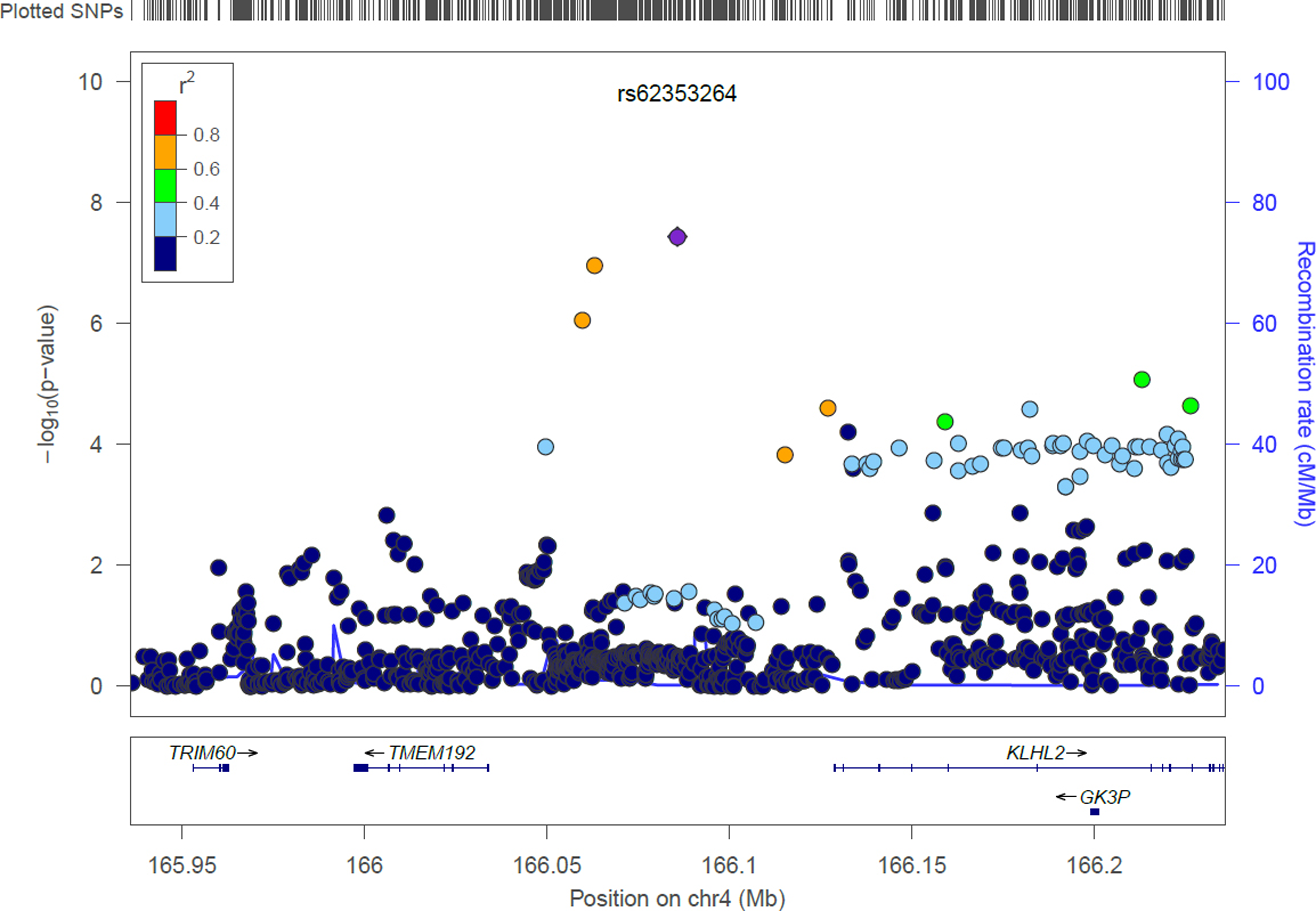

Supplement: Supplementary Figure 6 [file mp201649x6.tif]

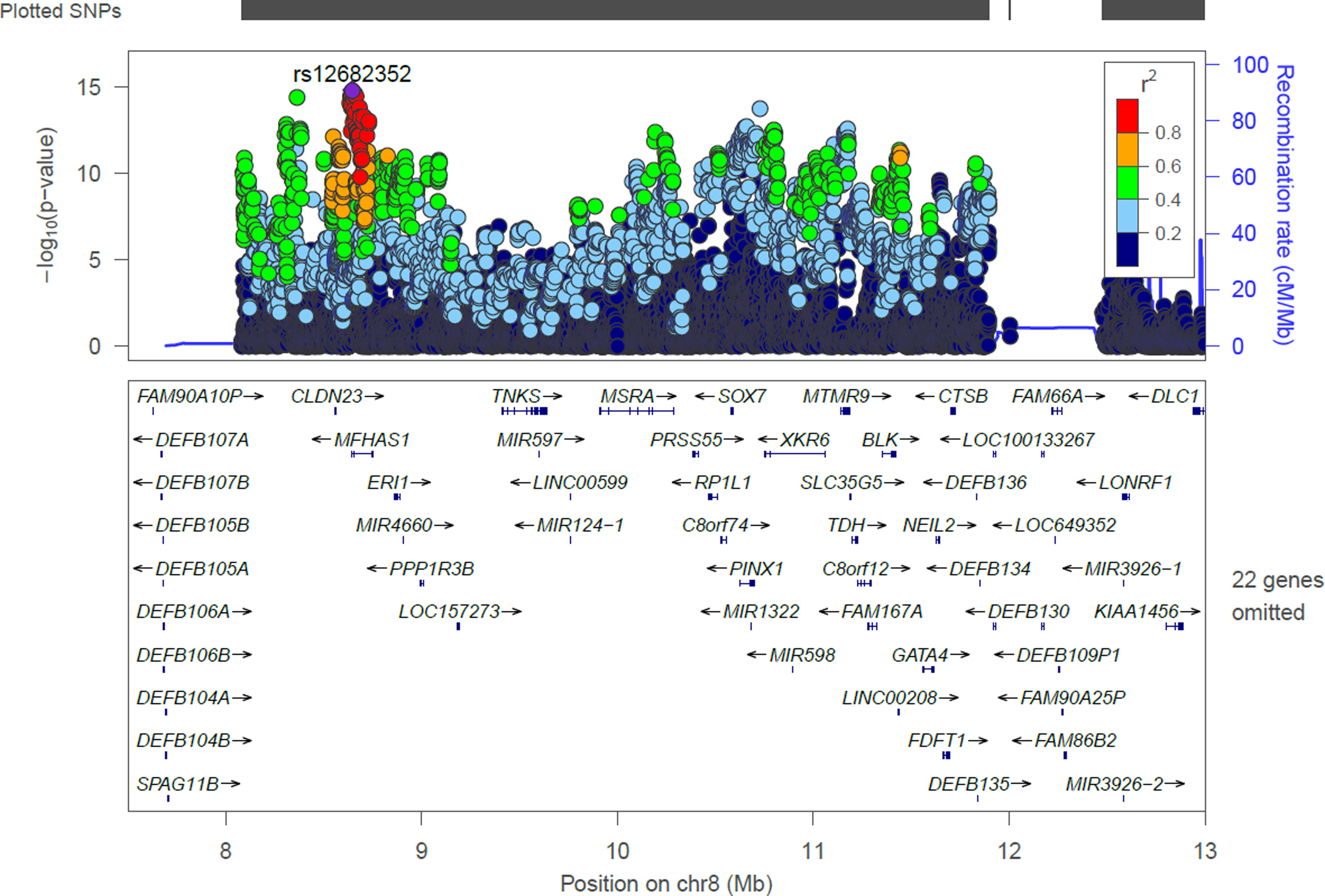

Supplement: Supplementary Figure 7 [file mp201649x7.tif]

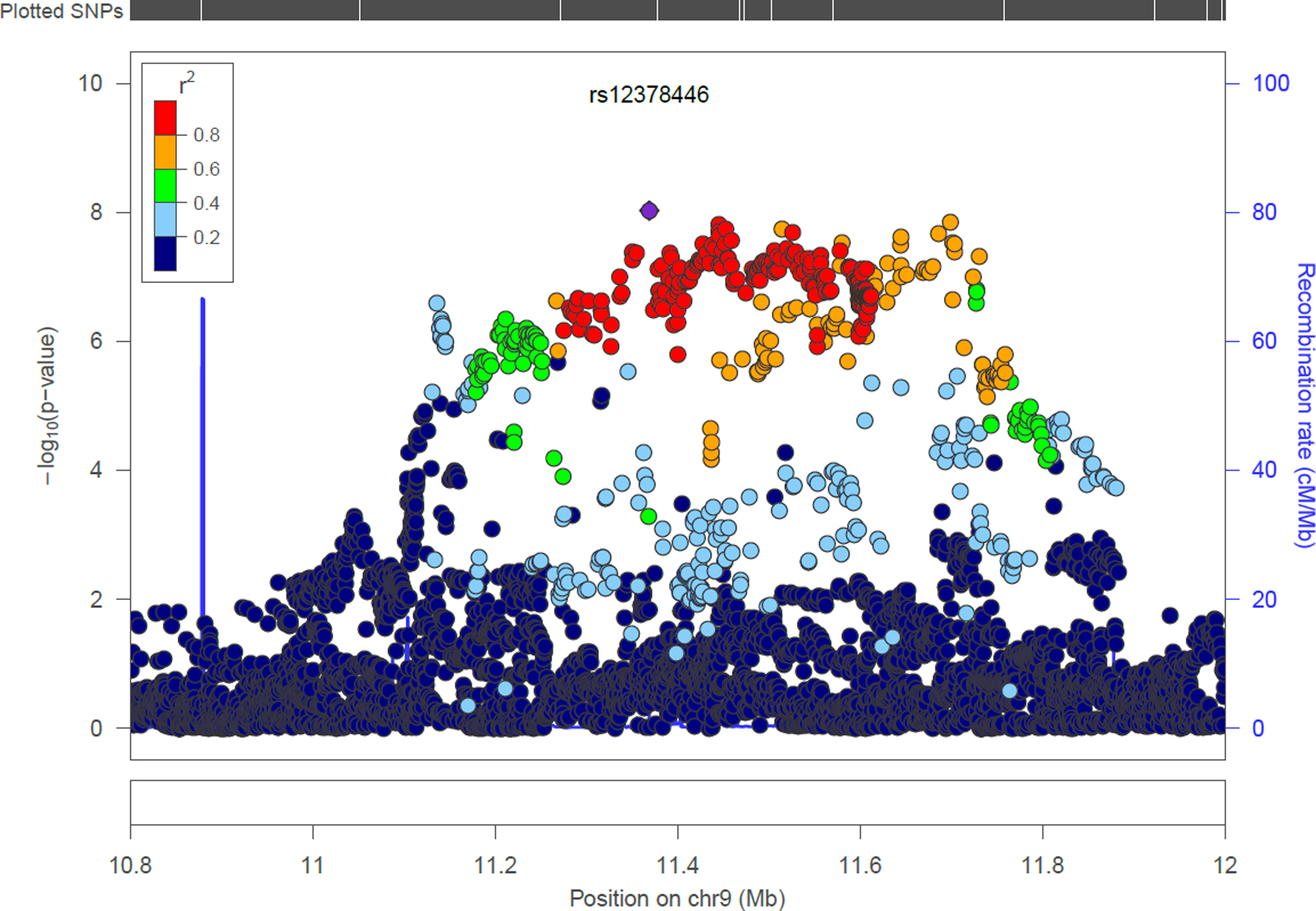

Supplement: Supplementary Figure 8 [file mp201649x8.tif]

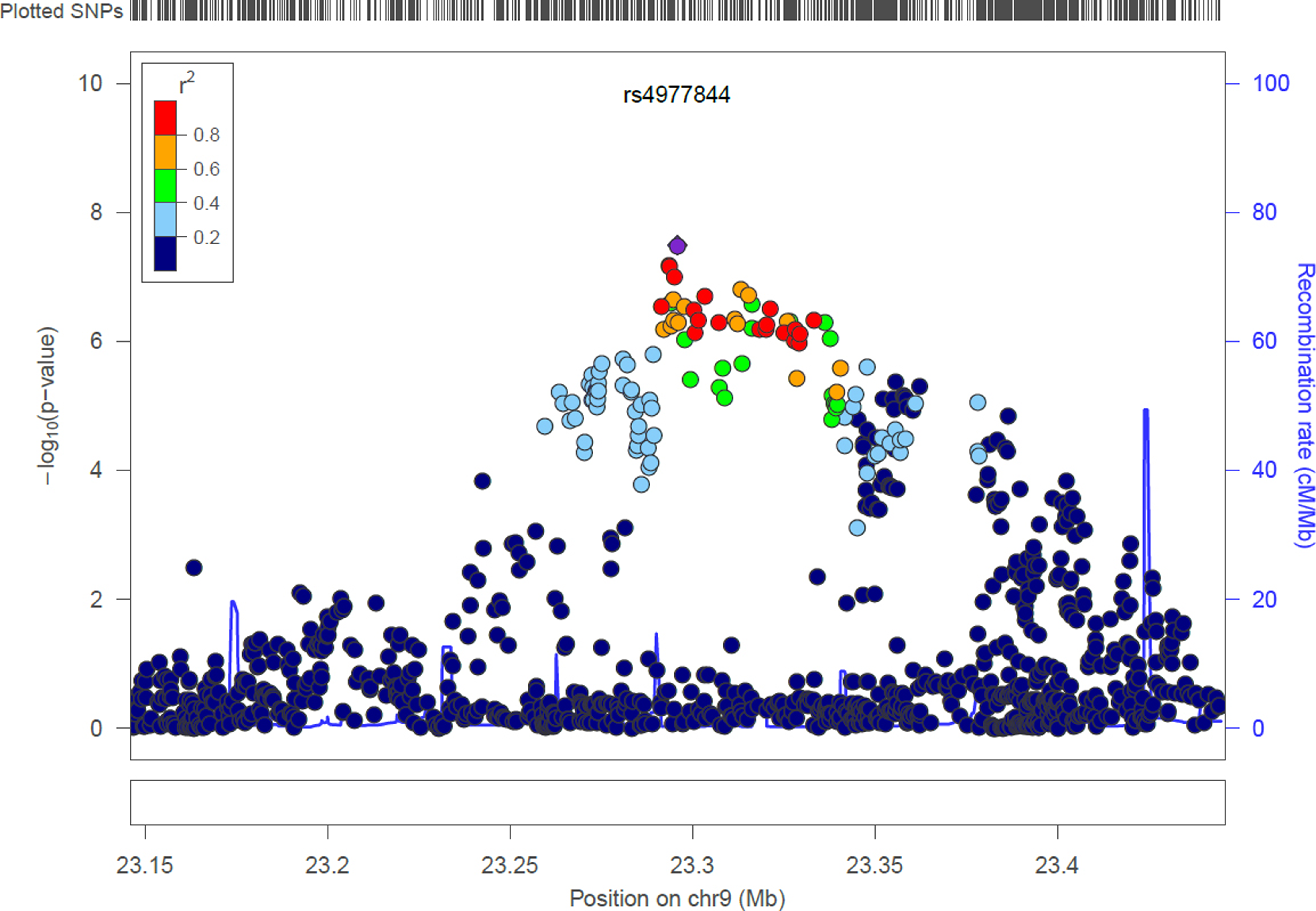

Supplement: Supplementary Figure 9 [file mp201649x9.tif]

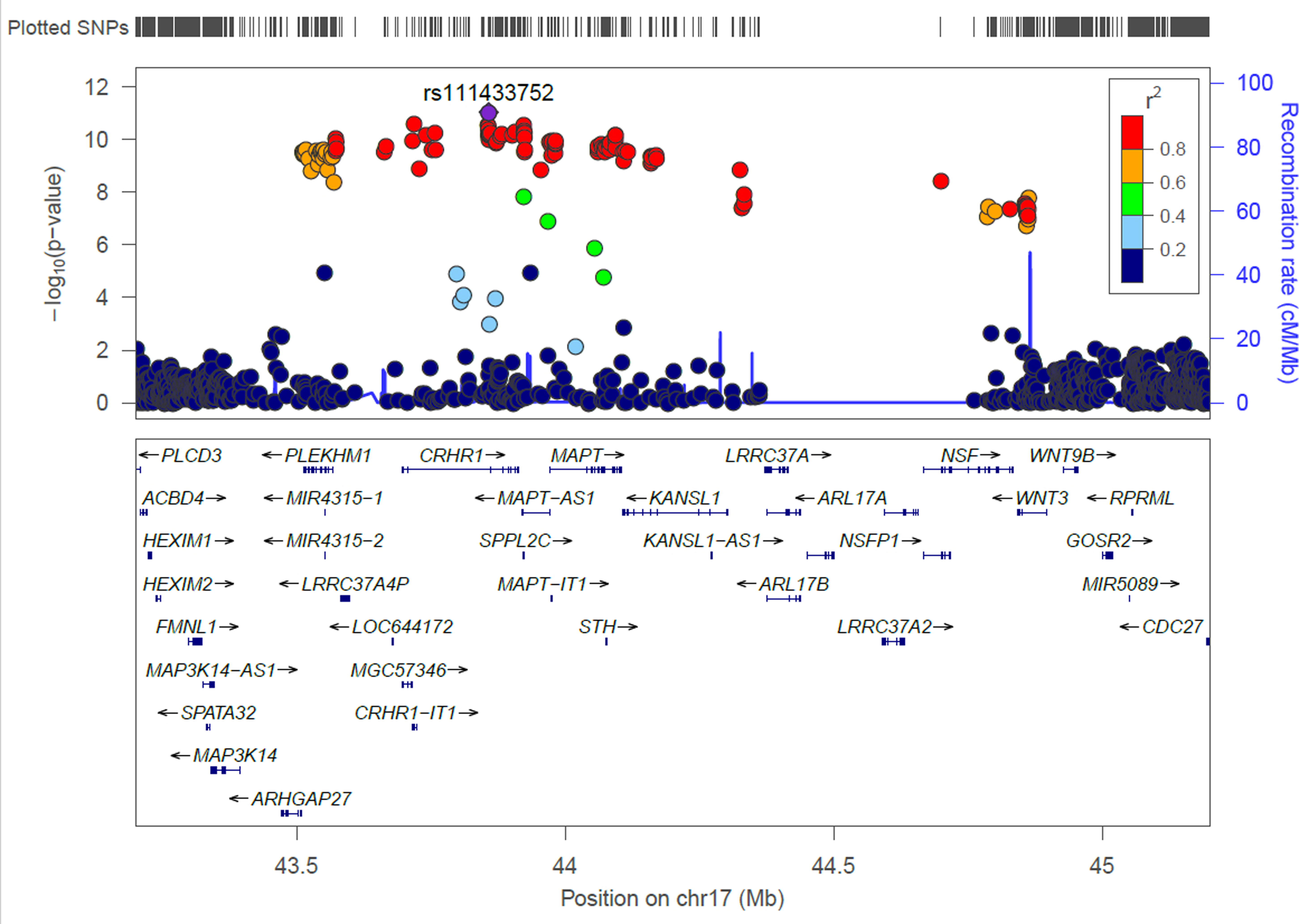

Supplement: Supplementary Figure 10 [file mp201649x10.tif]

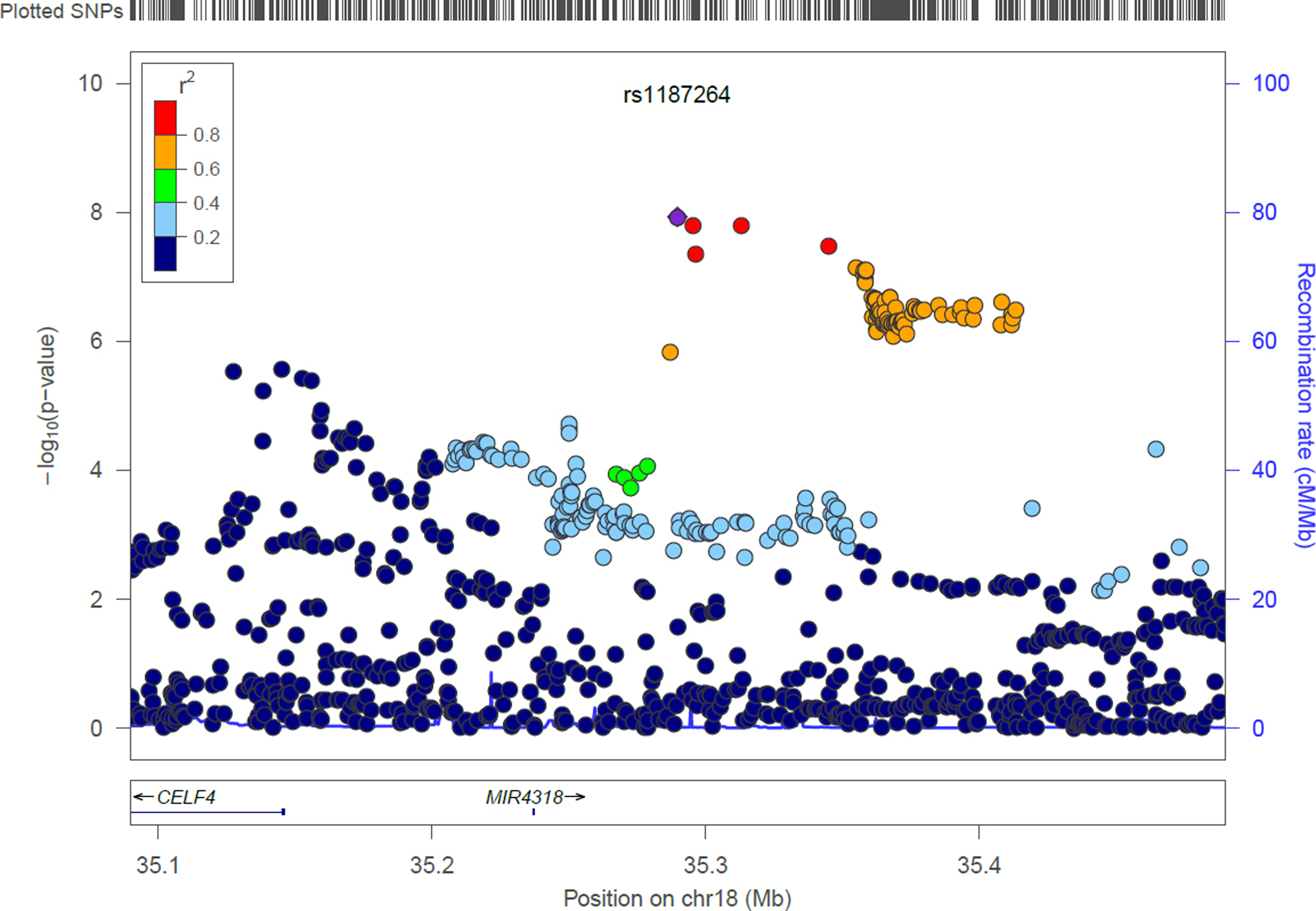

Supplement: Supplementary Figure 11 [file mp201649x11.tif]

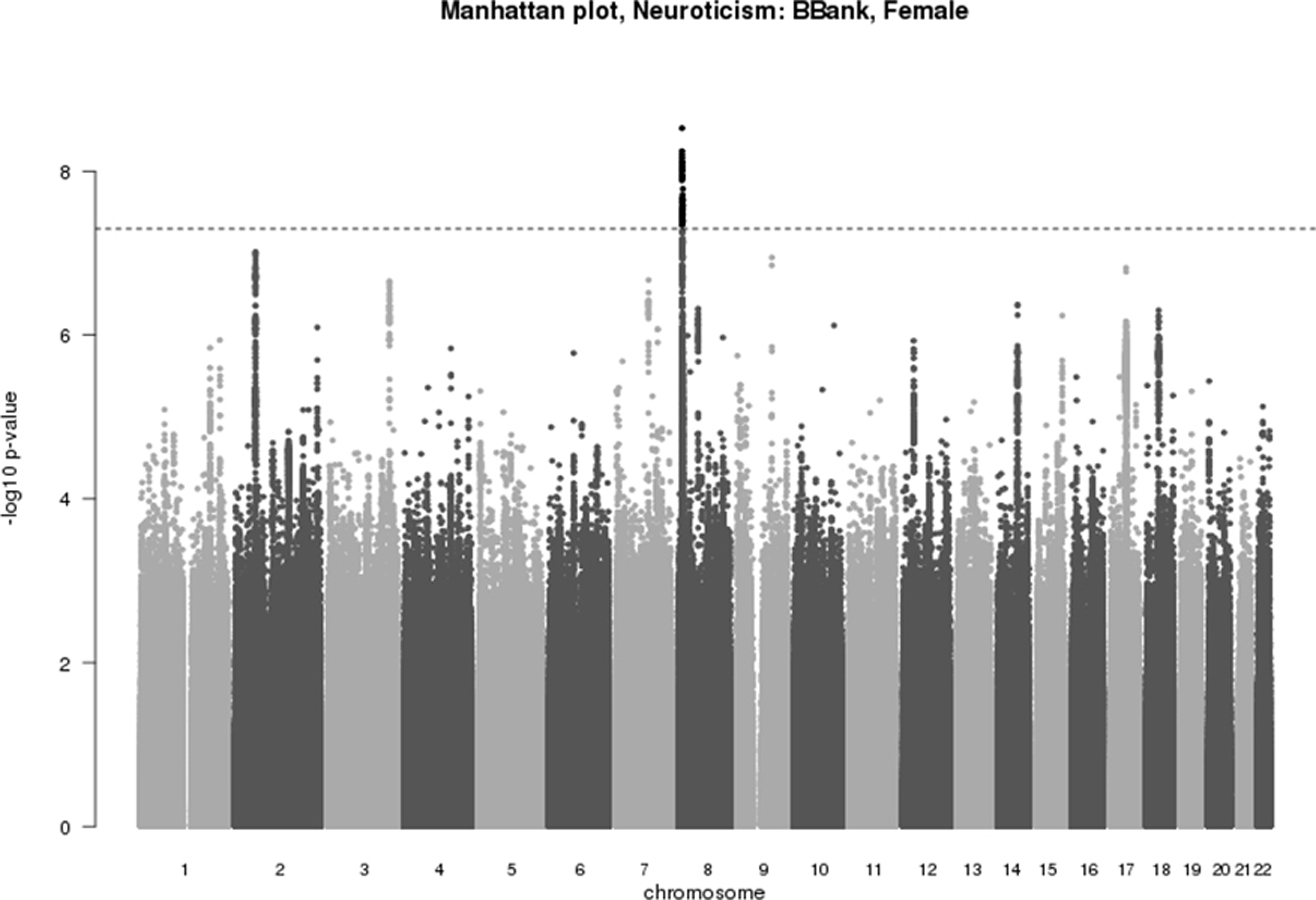

Supplement: Supplementary Figure 12 [file mp201649x12.tif]

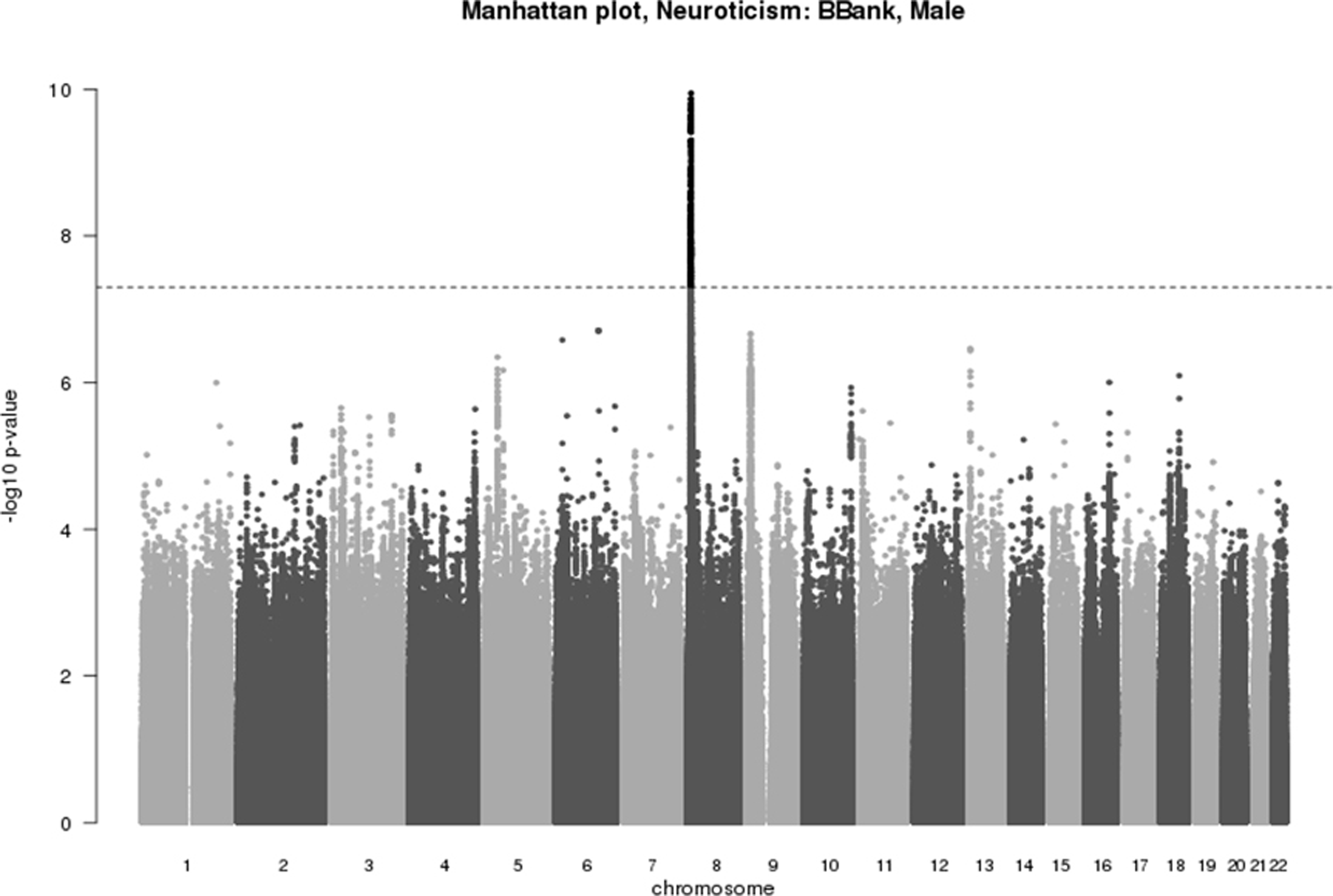

Supplement: Supplementary Figure 13 [file mp201649x13.tif]
