# Supplementary material for: Genome-wide analysis of over 106 000 individuals identifies 9 neuroticism-associated loci
Source: Mol Psychiatry. 2016 Apr 12;21(6):749–57. doi: 10.1038/mp.2016.49 (PMC4879189; doi:10.1038/mp.2016.49)
Supplement: Supplementary Tables [file mp201649x14.doc]

**Supplementary Material**

Figure S1. QQ plot for genome-wide association with neuroticism (n=91,370 UK Biobank participants only).

Figure S2. Manhattan plot (GWAS of N = 91,370 UK Biobank participants only).

Figure S3. Regional plots of genome-wide significant loci within the meta-analysis of UK Biobank, GS:SFHS and QIMR samples (figures S3a-S3i).

Figure S4. Manhattan plot for genome-wide association with neuroticism in UK Biobank, females only (n=47,196).

Figure S5. Manhattan plot for genome-wide association with neuroticism in UK Biobank, males only (n=44,174).

**Table S1. Eysenck Personality Questionnaire-Revised Short Form (EPQ-R-S) Neuroticism scale** .

|  |  | UK Biobank data-field |
| --- | --- | --- |
| 1 | Does your mood often go up and down? | 1920 |
| 2 | Do you ever feel 'just miserable' for no reason? | 1930 |
| 3 | Are you an irritable person? | 1940 |
| 4 | Are your feelings easily hurt? | 1950 |
| 5 | Do you often feel 'fed-up'? | 1960 |
| 6 | Would you call yourself a nervous person? | 1970 |
| 7 | Are you a worrier? | 1980 |
| 8 | Would you call yourself tense or 'highly strung'? | 1990 |
| 9 | Do you worry too long after an embarrassing experience? | 2000 |
| 10 | Do you suffer from 'nerves'? | 2010 |
| 11 | Do you often feel lonely? | 2020 |
| 12 | Are you often troubled by feelings of guilt? | 2030 |

**Table S2. Component loadings (on the first unrotated principal component), internal consistency reliabilities and variance explained from principal components anal**ysis of the twelve EPQ-R-S items.

|  | | **Full UK Biobank sample with neuroticism data** (N=401,695) | **Neuroticism GWAS sample**  (N=91,370) |
| --- | --- | --- | --- |
| Item factor loadings | 1. Does your mood often go up and down? | 0.68 | 0.62 |
|  | 2. Do you ever feel ‘just miserable’ for no reason? | 0.64 | 0.62 |
|  | 3. Are you an irritable person? | 0.52 | 0.64 |
|  | 4. Are your feelings easily hurt? | 0.59 | 0.63 |
|  | 5. Do you often feel ‘fed-up’? | 0.66 | 0.62 |
|  | 6. Would you call yourself a nervous person? | 0.61 | 0.63 |
|  | 7. Are you a worrier? | 0.63 | 0.62 |
|  | 8. Would you call yourself tense or ‘highly strung’? | 0.60 | 0.64 |
|  | 9. Do you worry too long after an embarrassing experience? | 0.58 | 0.63 |
|  | 10. Do you suffer from ‘nerves’? | 0.57 | 0.64 |
|  | 11. Do you often feel lonely? | 0.50 | 0.64 |
|  | 12. Are you often troubled by feelings of guilt? | 0.57 | 0.63 |
| Cronbach’s *α* | | 0.83 | 0.84 |
| % Variance explained by first unrotated principal component | | 36% | 33% |

**Table S3: Eight genome-wide significant associations for neuroticism within the** UK Biobank dataset.

| **Index SNP** | **Chr** | **Position** | **A1/A2** | **Frq** | **BETA (SE)** | **P** | **Associated regions** |
| --- | --- | --- | --- | --- | --- | --- | --- |
| rs2678897 | 2 | 58,169,418 | G/A | 0.391 | -0.088 (0.016) | 1.45x10^-8^ | 57,942,987- 58,484,172 |
| rs62353260 | 4 | 166,078,832 | A/G | 0.013 | 0.361 (0.066) | 3.78x10^-8^ | 166,049,663- 166,226,487 |
| rs140344078 | 7 | 7,700,640 | GT/G | 0.172 | -0.113 (0.020) | 1.43x10^-8^ | 7,683,347- 7,769,938 |
| rs12682352 | 8 | 8,646,246 | C/T | 0.475 | -0.12 (0.015) | 1.02x10^-15^ | 8,088,230- 11,922,801 |
| rs74311404 | 9 | 1,1506,513 | T/TAA | 0.220 | -0.103 (0.018) | 1.58x10^-8^ | 11,267,514- 11,810,796 |
| rs8081460 | 17 | 8,965,272 | A/G | 0.307 | -0.091 (0.016) | 2.65x10^-8^ | 8,964,004- 8,974,522 |
| rs549599956 | 17 | 44,247,164 | G/A | 0.232 | 0.106 (0.018) | 4.06x10^-9^ | 43,501,442- 44,863,133 |
| rs1187256 | 18 | 35,295,330 | T/C | 0.128 | 0.127 (0.023) | 2.16x10^-8^ | 35,287,090- 35,413,260 |

*Shown are LD-independent genome-wide significant SNP associations for neuroticism (sorted by genomic position according to UCSC hg19/NCBI Build 37). Column A1/A2 has the SNP alleles, with the first allele (a1) the reference allele for the frequency and BETA columns. Frq=frequency of allele 1. Chr and Position denote the location of the index SNP. BETA=linear regression coefficient for allele1, SE=standard error for BETA. Column 8 notes intersections of all genome-wide significant SNPs positions +/-500KB, with protein-coding genes based on GENCODE gene models (v19, file= gencode.v19.annotation.gtf filtered for feature_type=”gene”, gene_type="protein_coding" and gene_status="KNOWN", http://www.gencodegenes.org/releases/19.html).*

**Table S4. Genome-wide significant index SNPs from UK Biobank (or proxy where not available) within GS:SFHS and QIMR datasets.**

**proxy for rs26353260 (table S3); **proxy for rs74311404 (table S3); #proxy for rs549599956 (table S3).*
